# A De Novo Case of Floating Chromosomal Polymorphisms by Translocation in *Quasipaa boulengeri* (Anura, Dicroglossidae)

**DOI:** 10.1371/journal.pone.0046163

**Published:** 2012-10-03

**Authors:** Liyan Qing, Yun Xia, Yuchi Zheng, Xiaomao Zeng

**Affiliations:** 1 Department of Herpetology, Chengdu Institute of Biology, Chinese Academy of Sciences, Chengdu, People's Republic of China; 2 Graduate University of Chinese Academy of Sciences, Beijing, People's Republic of China; University of Arkansas, United States of America

## Abstract

Very few natural polymorphisms involving interchromosomal reciprocal translocations are known in amphibians even in vertebrates. In this study, thirty three populations, including 471 individuals of the spiny frog *Quasipaa boulengeri*, were karyotypically examined using Giemsa stain or FISH. Five different karyomorphs were observed. The observed heteromorphism was autosomal but not sex-related, as the same heteromorphic chromosomes were found both in males and females. Our results indicated that the variant karyotypes resulted from a mutual interchange occurring between chromosomes 1 and 6. The occurrence of a nearly whole-arm translocation between chromosome no. 1 and no. 6 gave rise to a high frequency of alternate segregation and probably resulted in the maintenance of the translocation polymorphisms in a few populations. The translocation polymorphism is explained by different frequencies of segregation modes of the translocation heterozygote during meiosis. Theoretically, nine karyomorphs should be investigated, however, four expected karyotypes were not found. The absent karyomorphs may result from recessive lethal mutations, position effects, duplications and deficiencies. The phylogenetic inference proved that all populations of *Q. boulengeri* grouped into a monophyletic clade. The mutual translocation likely evolved just once in this species and the dispersal of the one karyomorph (type IV) can explain the chromosomal variations among populations.

## Introduction

Very few natural polymorphisms involving interchromosomal reciprocal translocations are known in animal populations [1], [2]. The reasons are obvious because this kind of rearrangement can lead to reduction of fertility and reduced fitness for the carrier. Translocation heterozygotes will result in the formation of rings or chains of four chromosomes during meiosis and if they orientate with the adjacent centromeres passing to same poles (adjacent segregation) instead of to the opposite poles (alternate segregation), aneuploid gametes carrying either a duplication or a deficiency will be produced and would result in lower fecundity [3].

In amphibians, there are no reports of fixed translocation polymorphisms in natural populations but rare spontaneous translocations have been documented. Morescalchi (as cited in Chiarelli and Capanna, 1973, p326) [3] described a possible case of a simple translocation based on an analysis of oocyte lampbrush chromosomes from a female toad (*Pelodytes punctatus*). In 2004, Schmid et al. [4] observed a low frequency (2.7%) of a non-reciprocal translocation between autosomes 3 and 11 that were identified by the BrdU/dT replication banding patterns in aging cultured fibroblast cells of *Gastrotheca riobambae*. Schmid et al (2010). [5] found only three of 2,548 individuals (0.001%) of terraranan frogs that demonstrated a constitutional reciprocal translocation. Non-fixed mutual translocations have been found by Siqueira-Jr et al. (2004) [6] in *Haddadus binotatus*, also a species of terraranan frogs, however, few individuals were examined so translocation polymorphisms at a population level are not known.

**Figure 1:**
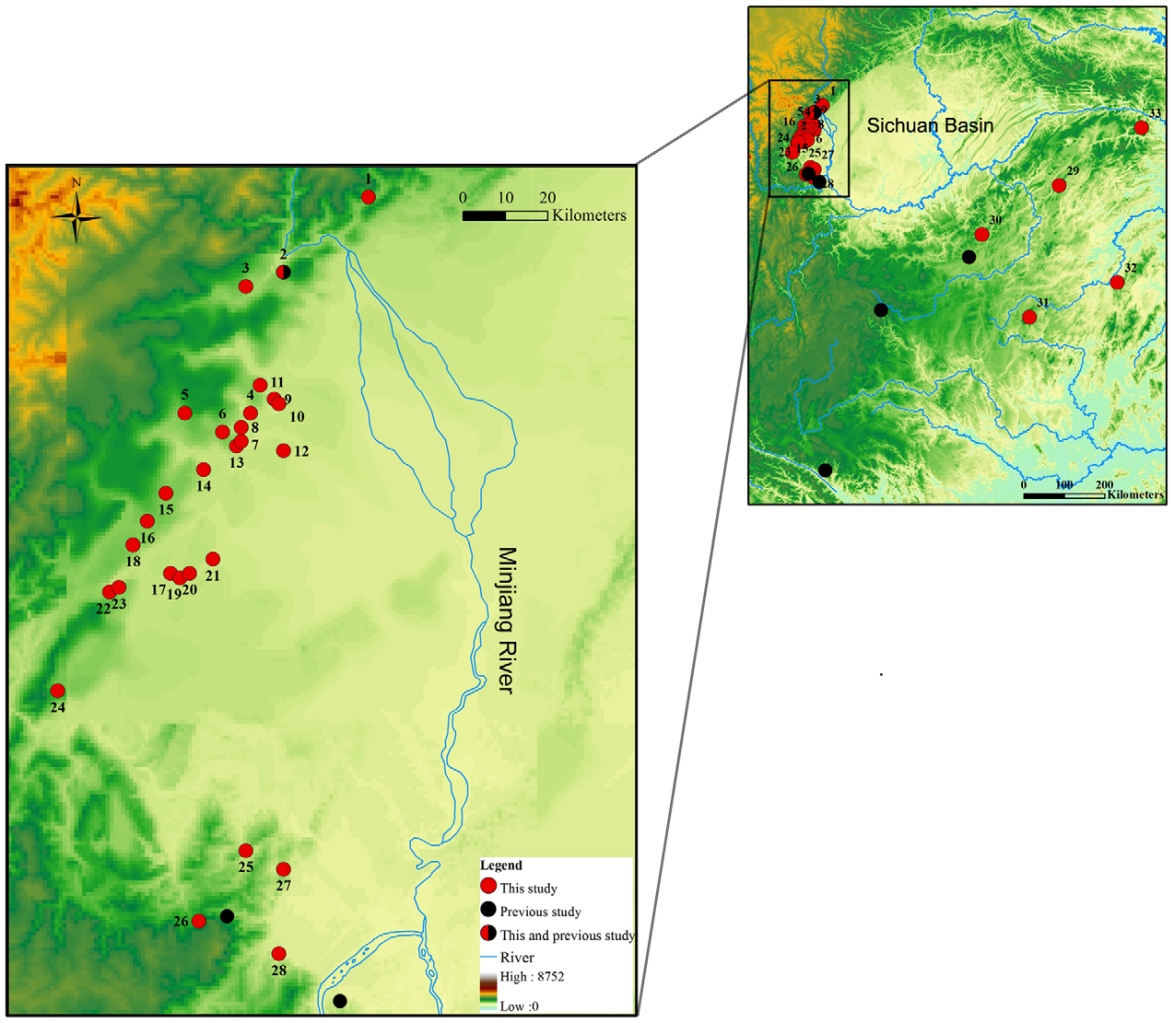
Map showing the sites of this and previous study around Sichuan Basin, China. The site names designated as in Table 1.

Recently, a fascinating case of interchange translocation polymorphisms was discovered in the Asian spiny frog, *Quasipaa boulengeri*. This species is widely distributed in low mountainous regions along the edges of Sichuan Basin and nearby areas in southern China [7] (Fig. 1). No heteromorphic chromosomes had been found in previous studies that examined several populations [8]–[13]. In a Xuankouzhen population, Wang (2006) [14] discovered two pairs of heteromorphic chromosomes in a single female and these chromosome pairs were homomorphic in males (3♂, 1♀, Table 1). He speculated that female sex-related heteromorphism exists, and the heteromorphic chromosomes were multiple sex chromosomes of a Z_1_W_1_Z_2_W_2_♀/Z_1_Z_1_Z_2_Z_2_♂ configuration. Further, he predicted that there exists a heterozygous reciprocal translocation between these two pairs of heteromorphic chromosomes.

**Table 1:** Synopsis of the karyomorph types.

Localities	Number of animals	Number of ♂/♀		♂					♀					
				I	II	III	IV	V	I	II	III	IV	V	References
Hongkouxiang, Dujiangyan City, Sichuan (1)	6	5	1	5	–	–	–	–	**–**	–	1	–	**–**	This study
* Xuankouzhen, Wenchuan Co., Sichuan (2)	8	1	7	–	–	–	1	–	1	–	–	6	**–**	This study
*Mt. Qingcheng, Sichuan (3)	13	3	10	2	–	–	1	–	5	–	–	5	**–**	This study
Wushanxiang, Dayi Co., Sichuan (4)	21	11	10	11	–	–	–	**–**	9	**–**	**–**	1	–	This study
Xilingzhen, Dayi Co., Sichuan (5)	10	6	4	5	–	–	1	–	4	–	**–**	**–**	**–**	This study
Xieyuanzhen-1, Dayi Co., Sichuan (6)	7	4	3	4	–	–	–	–	3	–	–	–	**–**	This study
Hemingxiang-1, Dayi Co., Sichuan (7)	3	2	1	2	–	–	–	–	1	–	–	–	–	This study
Hemingxiang-2, Dayi Co., Sichuan (8)	10	3	7	3	–	–	–	–	–	–	–	7	–	This study
*Jinxingxiang-1, Dayi Co., Sichuan (9)	16	11	5	10	–	–	1	–	2	–	–	3	–	This study
*Jinxingxiang-2, Dayi Co., Sichuan (10)	55	14	41	9	–	–	5	–	21	–	1	19	–	This study
Jinxingxiang-3, Dayi Co., Sichuan (11)	21	7	14	7	–	–		–	14	–	–		–	This study
*Gaotangsi, Dayi Co., Sichuan (12)	27	8	19	–	–	–	8	–	1	–	–	18	–	This study
Xinchangzhen, Dayi Co., Sichuan (13)	8	4	4	4	–	–	–	–	4	–	–	–	–	This study
Datongxiang, Qionglai Co., Sichuan (14)	16	9	7	9	–	–	–	–	7	–	–	–	–	This study
Shuikouzhen-2, Qionglai Co., Sichuan (15)	4	3	1	3	–	–	–	–	1	–	–	–	–	This study
Nanbaoxiang-1, Qionglai Co., Sichuan (16)	11	9	2	9	–	–	–	–	2	–	–	–	–	This study
Huojingzhen, Qionglai Co., Sichuan (17)	15	6	9	6	–	–	–	–	9	–	–	–	–	This study
Gaohezhen-2, Qionglai Co., Sichuan (18)	7	2	5	2	–	–	–	–	5	–	–	–	–	This study
Daozuoxiang-1, Qionglai Co., Sichuan (19)	29	4	25	4	–	–	–	–	25	–	–	–	–	This study
Daozuoxiang-2, Qionglai Co., Sichuan (20)	18	4	14	4	–	–	–	–	14	–	–	–	–	This study
Pinglezhen, Qionglai Co., Sichuan (21)	2	–	2	–	–	–	–	–	–	2	–	–	–	This study
Mt. Tiantai-1, Qionglai Co., Sichuan (22)	19	6	13	6	–	–	–	–	8	1	–	–	4	This study
Mt. Tiantai-2, Qionglai Co., Sichuan (23)	9	1	8	1	–	–	–	–	8	–	–	–	–	This study
Bifeng Valley, Yaan City, Sichuan (24)	11	6	5	6	–	–	–	–	5	–	–	–	–	This study
Huatouzhen, Jiajiang Co., Sichuan (25)	21	3	18	3	–	–	–	–	18	–	–	–	–	This study
Mt. Emei, Sichuan (26) Shuangfuzhen, Emeishan City, Sichuan (27)	14 26	7 8	7 18		7 8	– –	– –	– –	– –	7 18	– –	– –	– –	– –	This study This study
Luomuzhen, Emeishan City, Sichuan (28)	17	3	14		3	–	–	–	–	14	–	–	–	–	This study
Xinglongzhen, Youyang Co., Chongqing (29)	4	2	2		2	–	–	–	–	2	–	–	–	–	This study
Kuankuoshui, Suiyang, Guizhou (30)	14	7	7		7	–	–	–	–	7	–	–	–	–	This study
Leigongshan, Guizhou (31)	4	2	2		2	–	–	–	–	2	–	–	–	–	This study
Xuefengshan, Hunan (32)	14	7	7		7	–	–	–	–	7	–	–	–	–	This study
Hejiapingzhen, Changyang Co., Hubei (33)	11	5	6		5	–	–	–	–	6	–	–	–	–	This study
**Total**	471	173	298		156	–	–	17	–	230	3	2	59	4	This study
#Xuankouzhen, Wenchuan Co., Sichuan	4	3	1		3	–	–	–	–	–	–	–	1	–	Wang 2006
Mt. Emei & Leshang City, Sichuan	8	4	4		4	–	–	–	–	4	–	–	–	–	Chen et al. 1983
Mt. Emei, Sichuan	10	5	5		5	–	–	–	–	5	–	–	–	–	Wang et al. 1983
Mt. Emei, Sichuan	3	1	2		1	–	–	–	–	2	–	–	–	–	Wang 2006
Shuicheng Co., Guizhou	8	5	3		5	–	–	–	–	3	–	–	–	–	Zhang et al. 1997
Zunyi Co., Guizhou	?	?	?		?	–	–	–	–	?	–	–	–	–	Li and Hu 1999
Pingbian Co., Yunnan	3	1	2		1	–	–	–	–	2	–	–	–	–	Li and Hu 1996
Pingbian Co., Yunnan	?	?	?		?	–	–	–	–	?	–	–	–	–	Hu 2004

Roman numerals represent different karyomorphs with chromosome no.1/no.6: I, MM/mm; II, MM/mSt; III, MT/mm; IV, MT/mSt; V, MT/StSt (M =  large metacentric chromosome; m =  small metacentric chromosome; St =  large subtelocentric chromosome; T =  large telocentric chromosome).

“*” Populations with heteromorphic chromosomes in both males and females.

“#” Specimens from Mt. Qingchengshan reported by Wang (2006) are actually from Xuankouzhen (personal communication).

“?” The numbers of experimental animal are not shown in the references. Numbers in parentheses stand for the collecting localities.

To understand this rare karyotype, we have cytogenetically re-examined the species *Quasipaa boulengeri* at a population level since 2006. We examined 33 populations and 471 individuals. Our results revealed **a de novo case** of reciprocal translocation polymorphisms in amphibians that involves the most complicated multiple karyotypes so far known to exist in amphibians. Herein, we have reconstructed the phylogeny of the species *Q. boulengeri* based on DNA sequences of three mitochondrial genes (12S, 16S and COI) to test if the this species is monophyletic, and then we try to elucidate these polymorphic karyotypes in different populations, and to decipher the origin of the chromosomal polymorphisms between populations in this frog.

## Results

### Mitotic Chromosomes

**Figure 2:**
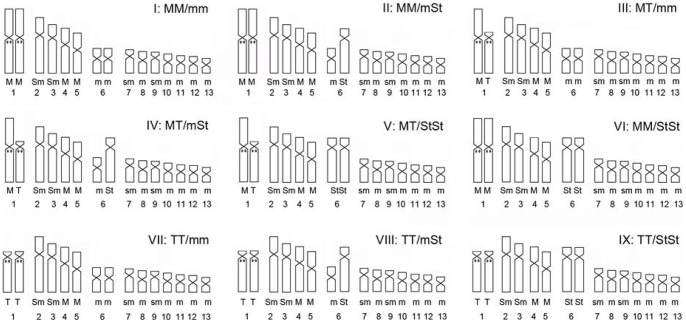
Idiograms of nine expected karyomorphs show morphological variations in no.1 and no. 6. Karyomorph type I, II, III, IV and V are present while type VI, VII, VIII and IX are absent in the investigated populations. All of the idiograms are based on the quantitative data of Table 5. Abbreviations designated as in Tables 1, 5. Black dots on the chromosomes show the 5S rDNA locations.

**Figure 3:**
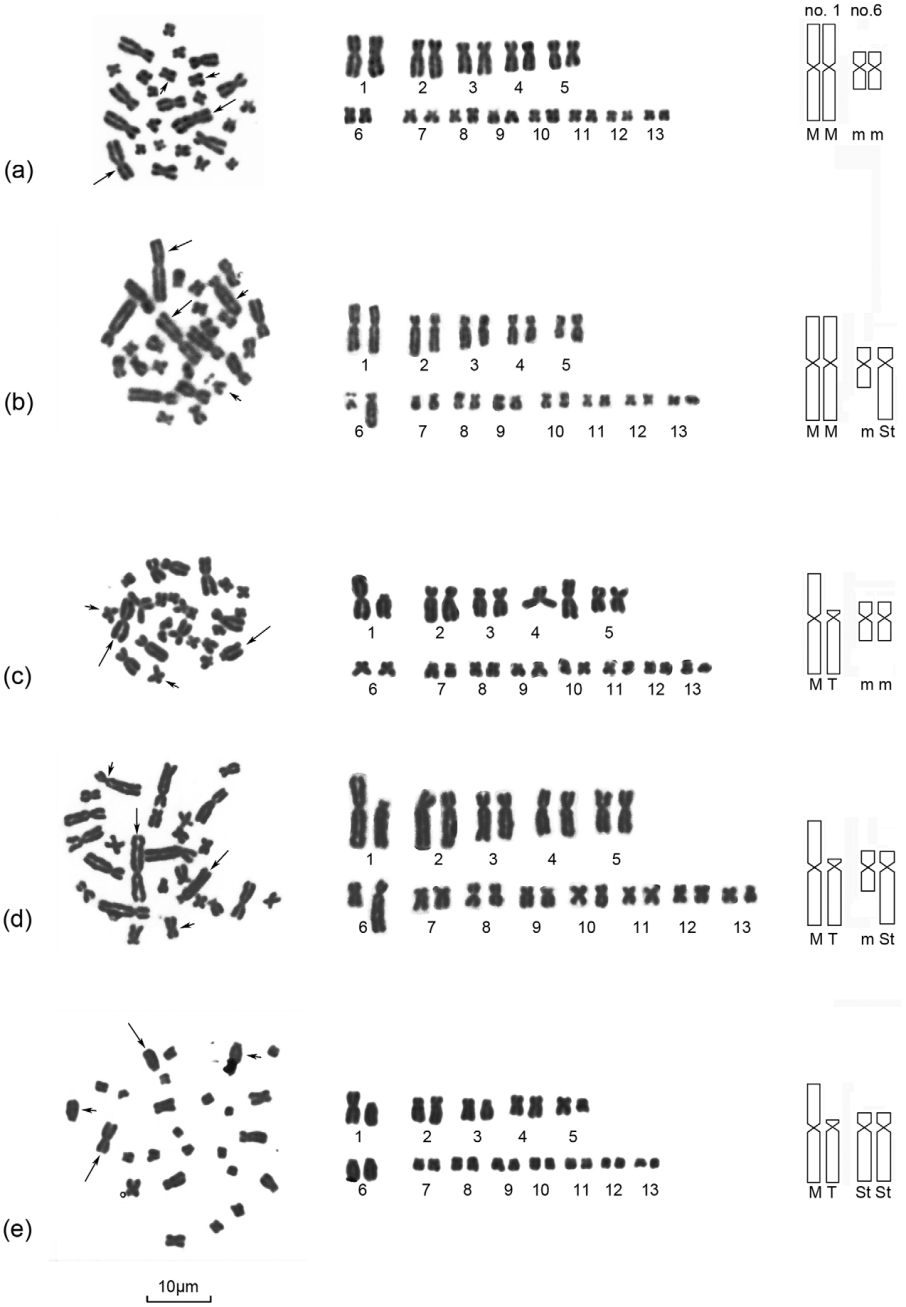
Mitotic metaphase and five different karyomorphs. Left row: mitotic metaphase; middle row: karyotypes; right row: idiograms of no.1 and no. 6. (a) Karyoype I obtained homomorphic biarmed chromosomes no. 1 (M/M) and no. 6 (m/m), the metaphase spread was taken from one male of Mt. Omei population; (b) Karyoype II had homomorphic biarmed chromosomes no. 1 (M/M), heteromorphic biarmed chromosomes no. 6 (m/St), the metaphase spread was taken from one female of Pinglezhen population; (c) Karyoype III was found to possess heteromorphic biarmed chromosomes no. 1 (M/T), homomorphic biarmed chromosomes no. 6 (m/m), the metaphase spread was taken from one female of Jinxingxiang-2 population; (d) Karyoype IV had heteromorphic biarmed chromosomes no. 1 (M/T), no. 6 (m/St), the metaphase spread was taken from one male of Gaotangsi population; (e) Karyoype V was characterised with heteromorphic chromosomes no. 1 (M/T), homomorphic biarmed chromosomes no. 6 (St/St), the metaphase spread was from one female of Mt. Tiantai-1 population. Long arrows indicated chromosomes no. 1; short arrows indicated chromosomes no. 6. Scale bar = 10 μm.

All specimens from all 33 populations had a diploid number of 2n = 26 chromosomes, and inter- and intrapopulation karyotype variations were found in several populations. At least five different karyomorphs (Type I–V) were observed in the pooled populations (Figs. 2, 3; Tables 1, 2). All karyotypic differences found in these populations were chromosome types that involved pairs no.1 and no.6.

**Table 2:** Constitution of the five karyomorphs.

no. Type	1–1	1–2	2	3	4	5	6–1	6–2	7	8	9	10	11	12	13
I	**M**	**M**	Sm	Sm	**M**	**M**	m	m	sm	m	sm	m	m	m	m
II	**M**	**M**	Sm	Sm	**M**	**M**	m	St	sm	m	sm	m	m	m	m
III	**M**	**T**	Sm	Sm	**M**	**M**	m	m	sm	m	sm	m	m	m	m
IV	**M**	**T**	Sm	Sm	**M**	**M**	m	St	sm	m	sm	m	m	m	m
V	**M**	**T**	Sm	Sm	**M**	**M**	St	St	sm	m	sm	m	m	m	m

Roman numerals (I∼V) represent five karyomorph types.

The bold mumbers and letters indicate the homologues of no. 1 and no. 6 and their cenromere position, respectively.

M =  large metacentric chromosome; m =  small metacentric chromosome; Sm =  large submetacentric chromosome;

sm =  small submetacentric chromosome; St =  large subtelocentric chromosome; T =  large telocentric chromosome.

Variations exist in the types of chromosome no. 1 and no. 6.

#### Type I (MM/mm)

The normal karyotype. Chromosome numbers 1 and no. 6 consisted of two large homomorphic metacentric (MM) chromosomes and two small homomorphic metacentric (mm) chromosomes, respectively (Figs. 2-I, 3a).

#### Type II (MM/mSt)

This type was characterized by having a large pair of homomorphic metacentric no. 1 (MM) and a pair of heteromorphic no. 6 chromosomes, which consisted of a small metacentric chromosome (m) and a large subtelocentric chromosome (St) (Figs. 2-II, 3b).

#### Type III (MT/mm)

Heteromorphic chromosome no. 1 and homomorphic chromosome no. 6 comprised type III karyomorph. The first chromosome pair was composed of a large metacentric (M) and a large telocentric chromosome (T). And the latter chromosome pair consisted of two homomorphic small metacentric chromosomes (mm) (Figs. 2-III, 3c).

#### Type IV (MT/mSt)

This type was also designated as translocation heterozygotes. It has two pairs of heteromorphic chromosomes (no. 1 and no. 6). Chromosome no. 1 is comprised of a large metacentric chromosome (M) and a large telocentric chromosome (T). Chromosome no. 6 consisted of a small metancentric chromosome (m) and a large subtelocentric chromosome (St) (Figs. 2-IV, 3d).

#### Type V (MT/StSt)

Chromosome no. 1 was heteromorphic with a large metacentric chromosome (M) and a large telocentric chromosome (T). The no. 6 was homomorphic with two large subtelocentric chromosomes (StSt) (Figs. 2-V, 3e).

Karyomorphs Type I (MM/mm) and Type IV (MT/mSt) are the most (82.0%) and second most (16.1%) common karyomorphs in *Quasipaa boulengeri*. The other three karyomorphs only made up about 1.8% of the karyomorphs (Table 3).

**Table 3:** Karyotype frequencies in each and pooled populations.

Localities	♂+♀				
	I	II	III	IV	V
Hongkouxiang, Dujiangyan City, Sichuan (1)	83.3%	–	16.7%	–	–
*Xuankouzhen, Wenchuan Co., Sichuan (2)	12.5%	–	–	87.5%	–
Mt. Qingcheng, Sichuan (3)	53.8%	–	–	46.2%	–
Wushanxiang, Dayi Co., Sichuan (4)	95.2%	–	–	4.8%	–
Xilingzhen, Dayi Co., Sichuan (5)	90.0%	–	–	10.0%	–
Xieyuanzhen-1, Dayi Co., Sichuan (6)	100.0%	–	–	–	–
Hemingxiang-1, Dayi Co., Sichuan (7)	100.0%	–	–	–	–
*Hemingxiang-2, Dayi Co., Sichuan (8)	30.0%	–	–	70.0%	–
Jinxingxiang-1, Dayi Co., Sichuan (9)	75.0%	–	–	25.0%	–
Jinxingxiang-2, Dayi Co., Sichuan (10)	54.5%	–	1.8%	43.6%	–
Jinxingxiang-3, Dayi Co., Sichuan (11)	100.0%	–	–	–	–
*Gaotangsi, Dayi Co., Sichuan (12)	3.7%	–	–	96.3%	–
Xinchangzhen, Dayi Co., Sichuan (13)	100.0%	–	–	–	–
Datongxiang, Qionglai Co., Sichuan (14)	100.0%	–	–	–	–
Shuikouzhen-2, Qionglai Co., Sichuan (15)	100.0%	–	–	–	–
Nanbaoxiang-1, Qionglai Co., Sichuan (16)	100.0%	–	–	–	–
Huojingzhen, Qionglai Co., Sichuan (17)	100.0%	–	–	–	–
Gaohezhen-2, Qionglai Co., Sichuan (18)	100.0%	–	–	–	–
Daozuoxiang-1, Qionglai Co., Sichuan (19)	100%	–	–	–	–
Daozuoxiang-2, Qionglai Co., Sichuan (20)	100.0%	–	–	–	–
Pinglezhen, Qionglai Co., Sichuan (21)	–	100.00%	–	–	–
Mt. Tiantai-1, Qionglai Co., Sichuan (22)	73.7%	5.3%	–	–	21.1%
Mt. Tiantai-2, Qionglai Co., Sichuan (23)	100.0%	–	–	–	–
Bifeng Valley, Yaan City, Sichuan (24)	100.0%	–	–	–	–
Huatouzhen, Jiajiang Co., Sichuan (25)	100.0%	–	–	–	–
Mt. Omei, Sichuan (26)	100.0%	–	–	–	–
Shuangfuzhen, Omeishan City, Sichuan (27)	100.0%	–	–	–	–
Luomuzhen, Omeishan City, Sichuan (28)	100.0%	–	–	–	–
Xinglongzhen, Youyang Co., Chongqing (29)	100.0%	–	–	–	–
Kuankuoshui, Suiyang, Guizhou (30)	100.0%	–	–	–	–
Leigongshan, Guizhou (31)	100.0%	–	–	–	–
Xuefengshan, Hunan (32)	100.0%	–	–	–	–
Hejiapingzhen, Changyang Co., Hubei (33)	100.0%	–	–	–	–
Total	82.0%	0.6%	0.4%	16.1%	0.8%

Locality names and numbers are consisted with those in Table 1;

Roman numerals (I∼IX) represent different karyotypes;

“*” Populations with more than 50% type IV.

### 5S rDNA FISH Karyotypes

**Figure 4:**
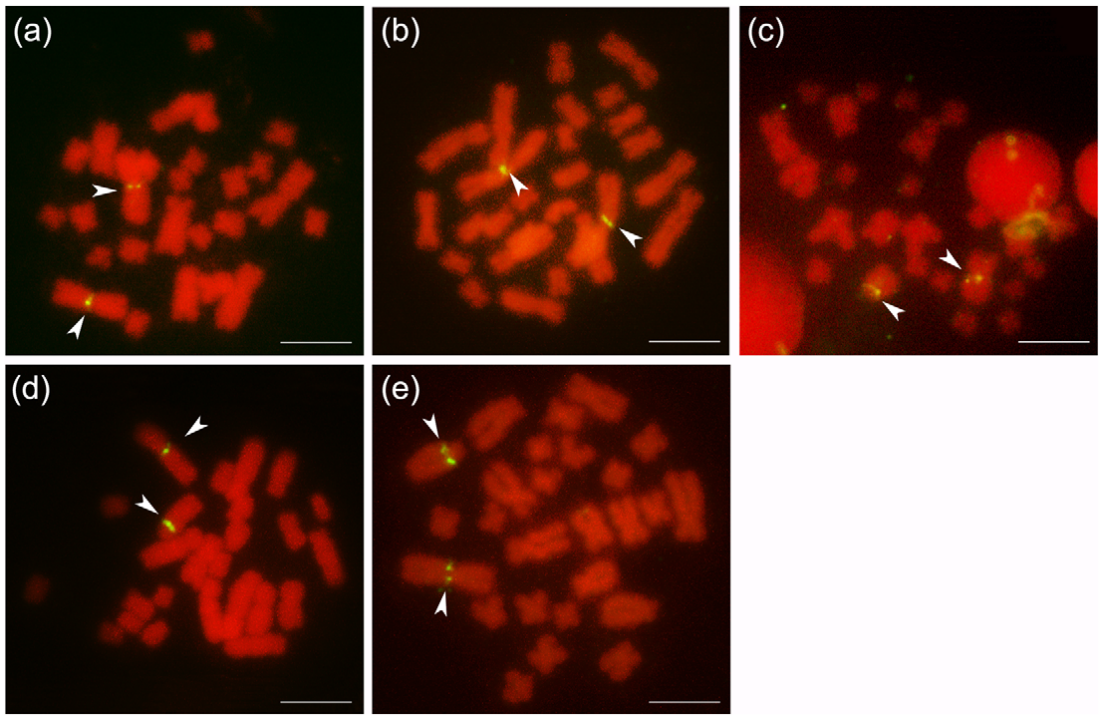
In situ hybridization of 5S rDNA to mitotic metaphase chromosomes. (a) Karyoype I, 5S rDNA sites were located on homomorphic no. 1 (M/M) chromosomes, the metaphase spread was taken from one male of Mt. Tiantai-1 population; (b) Karyoype II, 5S rDNA signals were found on homomorphic no. 1 (M/M) chromosomes, the metaphase spread was taken from one female of Mt. Tiantai-1 population; (c) Karyoype III, 5S rDNA loci were observed on heteromorphic chromosome no. 1 (M/T), the metaphase spread was taken from one female of Hongkouxiang population; (d) Karyoype IV, karyotype, 5S rDNA distributed on heteromorphic chromosomeno. 1 (M/T), the metaphase spread was taken from one male of Xilingzhen population; (e) Karyoype V, karyotype, 5S rDNA signals were investigated on heteromorphic chromosome no. 1 (M/T), the metaphase spread was taken from one female of Mt. Tiantai-1 population. Arrow heads indicated the 5S rDNA signals. Scale bar  = 5 μm.

In all five karyomorphs, 5S rDNA sites were located on no.1 homologues regardless the karyotypic morphology (Figs. 2, 4).

#### Type I

5S rDNA were detected near the centromeric region of the largest homomorphic chromosome pair (no. 1, MM) (Figs. 2-I, 4a). No signal was found on other homormophic chromosomes.

#### Type II

Even with one pair of heteromorphic chromosome no. 6, 5S rDNA was only found in the centromeric region of chromosome no. 1 (MM) (Figs. 2-II, 4b).

#### Type III

5S rDNA sites were distributed in the centromeric region of a large metancentric chromosome (M) and a large telocentric chromosome (T) (Figs. 2-III, 4c). Besides the chromosome size, the location of 5S rDNA provided further proof that these two heteromorphic chromosomes were homologous pairs composed of chromosome no. 1 (MT).

#### Type IV

In this karyomorph, 5S rDNA signals were also observed close to the centromere of a pair of heteromorphic chromosomes no. 1 (MT) like in karyotype III (Figs. 2-IV, 4d).

#### Type V

5S rDNA positions in this karyotype were at exactly the same location of no. 1 like type III and type IV (Figs. 2-V, 4e).

In conclusion, combined with the chromosome size, 5S rDNA can be used as a good marker to identify the homologous chromosome 1 and to facilitate the pairing of the heteromorphic homologues in *Quasipaa boulengeri*.

### Comment on “sex chromosomes”

Relying on his limited sample of three males and one female, Wang (2006) [14] hypothesized that a multiple sex-chromosome system exists in the Xuankouzhen population of *Quasipaa boulengeri*. No heteromorphic chromosomes were detected in the male individuals but in the single female, two pairs of heteromorphic chromosomes (no.1/no.6 in this study) were demonstrated. Thus, Wang speculated that the heteromorphic chromosomes were a demonstration of a Z_1_W_1_Z_2_W_2_♀/Z_1_Z_1_Z_2_Z_2_♂ type of sex determination.

Our present study revealed that the heteromorphic chromosomes (no. 1/no. 6) in the Xuankouzhen population of *Quasipaa boulengeri* represent an autosomal, rather than sex-associated heteromorphism. We re-collected specimens from Xuankouzhen (1♂, 7♀), and the same heteromorphic chromosomal pairs, as reported by Wang (2006) [14], were found in one male and six females (Table 1). Subsequent analysis of four other populations has shown a similar situation to Xuankouzhen. In Gaotangsi (8♂ of 27, 18♀ of 27) and Jinxingxiang-2 (5 ♂ of 55, 19♀ of 55) populations, the male and female rates involving heteromorphic chromosomes were both higher (Table 1). Obviously, the heteromorphic chromosomes are not related to sex heteromorphism and do demonstrate autosomal heteromorphic variation.

### Phylogenetic analysis

The results of the separate analyses based on different data sets were mostly mutually compatible. The alignment for the COI fragment was straightforward. But the alignment of several loop regions of the rRNA genes was ambiguous, and therefore, 68 and 7 sites with questionable homology of the rRNA+COI and COI+rRNA alignments were excluded from the rest of the analysis, respectively.

**Figure 5:**
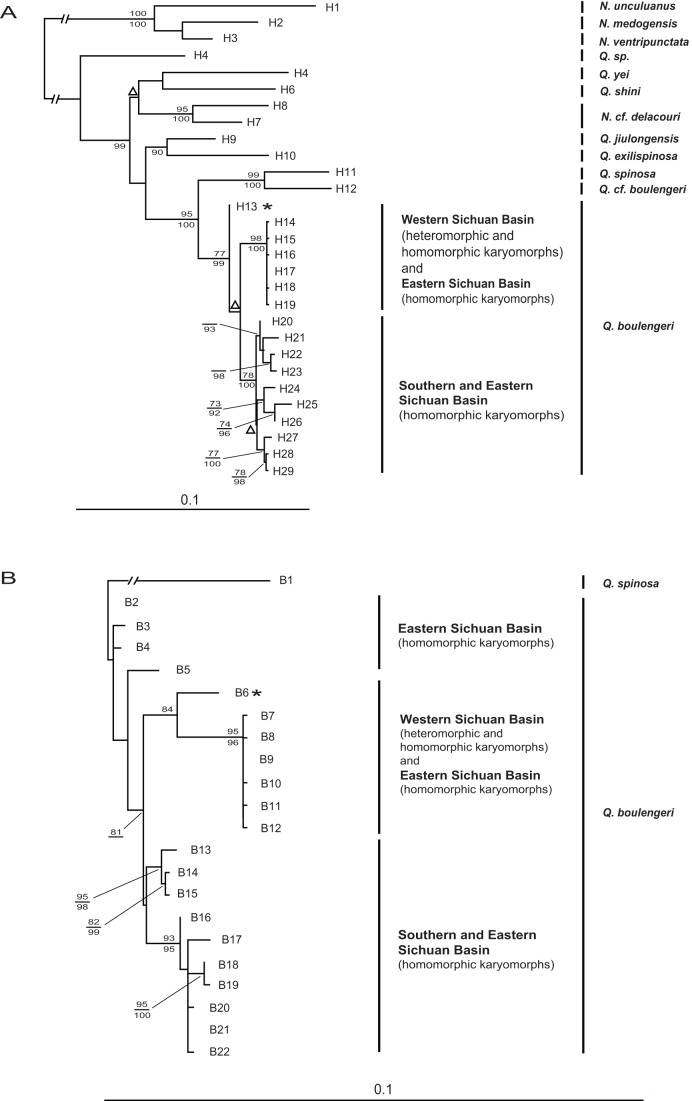
Phylogenetic trees of *Q. boulengeri* inferred from ML analyses of the three-partition mitochondrial rRNA+COI data set (A) or COI+rRNA data set (B). Numbers beside nodes are ML bootstrap proportions ≥70 (above branches) and Bayesian posterior probabilities ≥90 (under branches). All rRNA+COI haplotypes contained the 12S and 16S sequences, and all COI+rRNA haplotypes contained the COI sequence. Triangles indicate nodes that are not supported in the Bayesian analysis. Asterisk represents *Q. robertingeri* which is thought to be the synonym of *Q. boulengeri* (Che et al., 2009).

The rRNA+COI data set had 29 haplotypes and 1456 nucleotide sites (Table S1; Fig. 5A). In both ML and Bayesian analysis, all *Quasipaa boulengeri* samples formed a well supported clade with *Q. robertingeri* which was resolved as a synonym of the former [15]. Futhermore, the specimens possessing heteromorphic and homomorphic karyomorphs from Western Sichuan Basin belonged to a strongly support clade with extremely low sequence diversity. The COI+rRNA data set had 22 haplotypes and 1517 sites, and the Bayesian analyses with different model parameters produced an identical topology and similar posterior probabilities. Similarly, on both ML and Bayesian trees, samples from Western Sichuan Basin belonged to a highly supported clade with extremely low diversity(Table S1; Fig. 5B).

## Discussion

### Evidence for a reciprocal translocation

Reciprocal translocations can be identified using relative length and centromeric indices of the chromosomes [14], [16]. We measured and compared the chromosome arm lengths of hetero- and homomorphic chromosomes no.1 and no.6 which were chosen from the karyotypes of different individuals in several populations (Table 4), our results coincided with Wang's which were based on only one individual from a single population [14].

**Table 4:** The lengths of four hetermorphic chromosomes in ten cells(μm).

Chrom. No\Cell no.	1	2	3	4	5	6	7	8	9	10	Mean
1−1	5.38	9.07	7.60	9.58	7.19	6.02	5.02	5.71	4.70	4.99	
1−2	3.13	5.82	4.45	6.46	4.25	3.60	3.40	3.91	3.17	3.46	
6−1	2.24	3.00	3.03	3.38	2.81	2.39	2.06	2.05	2.00	2.15	
6−2	3.36	6.48	5.08	6.59	4.98	4.30	3.63	3.49	3.25	4.02	
(1−1)+(1−2)+(1−1)+(1−2)	14.11	24.37	20.17	26.01	19.23	16.31	14.12	15.16	13.11	14.62	17.72
[(1−1)+(6−1)]×2	15.25	24.14	21.26	25.92	20.00	16.80	14.16	15.52	13.39	14.28	18.07

The ten cells are respectively from ten individuals (5♀, 5♂) with karyotype IV from different populations. The T test is made between the sum of the length of the four heteromorphic chromosomes from chromosome no. 1 and no. 6 designed as (1−1)+(1−2)+(1−1)+(1−2) and the sum of the length of the two normal homologues designed as [(1−1)+(6−1)]×2, p>0.05. “1−1” and “1−2” refer to the length of two homologues of chromosome no. 1, respectively. “6−1” and “6−2” represent the length of two homologues of chromosome no.6, respectively.

It is important to know how the four homologues of the two pairs of translocated chromosomes match in a pair. Wang (2006) analyzed R-bands of the heteromorphic karyotypes and showed that the largest metacentric (M) and the largest telocentric chromosome (T) are a homologous pair composing the no. 1 chromosomes, while the other two heteromorphic chromosomes (m and St) are homologous chromosomes no. 6 [14]. Usually, such multiple bandings as R-bands are not definitive and can result in ambiguous assignments [17], [18]. In the present study, 5S rDNA FISH clearly demonstrates that a reciprocal translocation exists between heteromorphic chromosomes no. 1 and no. 6. By comparing the five hetero- and homomorphic karyotypes in different populations of *Quasipaa boulengeri*, 5S rDNA markers were separately located on the long arm of the largest metacentric as well as the largest telocentric chromosome, which provides strong evidence that these two heterochromosomes exactly match homologous chromosome pair of no. 1 (Fig. 4).

### Translocation polymorphisms

**Figure 6:**
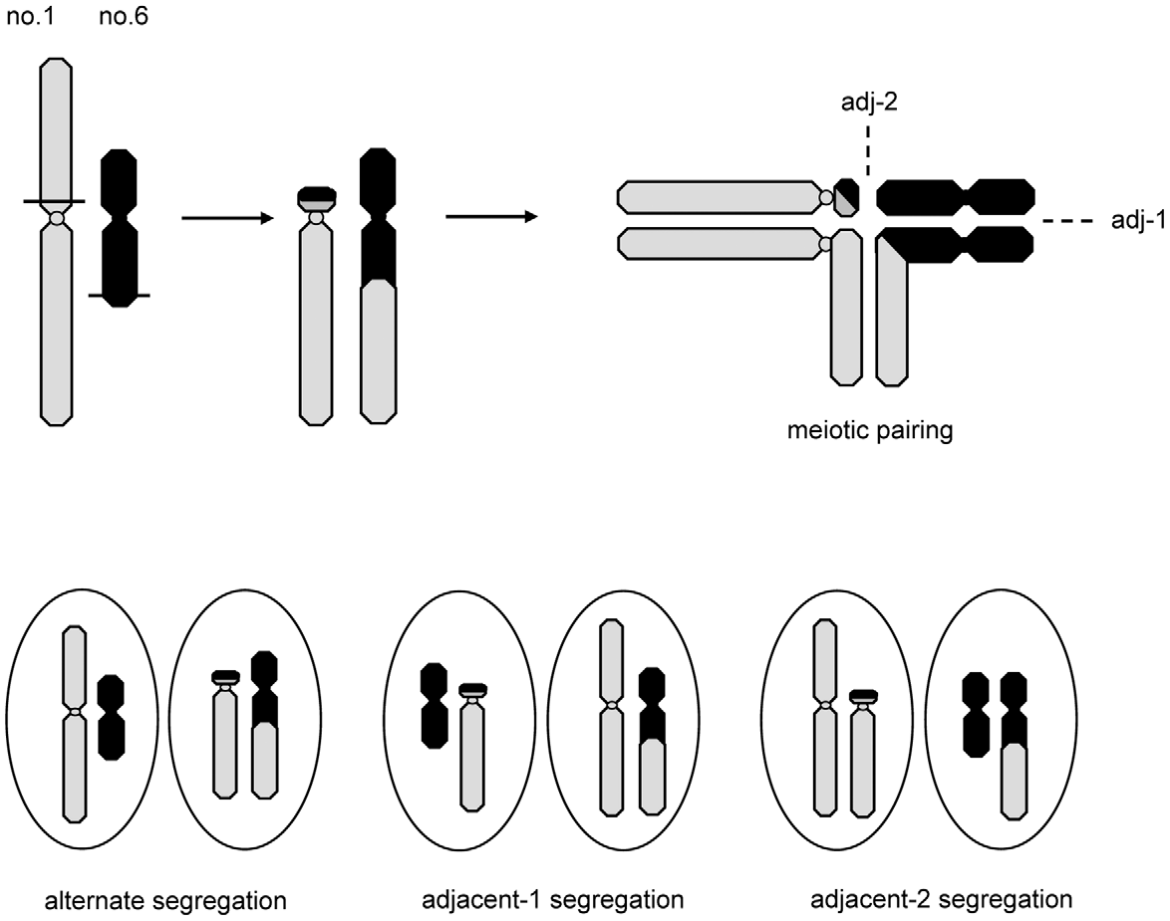
Hypothetical segregation of reciprocal translocation involving chromosome 1 and 6 during meiosis. Alternate segregation results in production of normal or balanced chromosomes. The separation of homologous centromeres (adjacent-1) or nonhomologous centromeres (adjacent-2) results in production of gametes with unbalanced chromosome. Horizontal lines represent the breakpoints.

The polymorphic karyotypes within and between different populations of *Quasipaa boulengeri*, are likely caused by a mutual translocation involving alternate and adjacent segregation in meiosis. A translocation heterozygote (type IV, MT/mSt) is expected to form a quadrivalent during meiosis. Three segregation modes (alternate, adjacent-1, and adjacent-2) could produce six types of gametes [19], i.e. M/m and T/St for alternate, M/St and m/T for adjacent-1, M/T and m/St for adjacent-2 modes (Fig. 6). If all these gametes are functional, 19 karyologically different offspring are expected to be observed in populations of this species. In the present study, however, based on the analysis of 471 individuals from 33 populations (Table 1), we have only observed five karyotypes (i.e. type I, II, III, IV, and V). They represent, respectively, MM/mm (I), MM/mSt (II), MT/mm (III), MT/mSt (IV), and MT/StSt (V) chromosomal pairs. These combinations can be attributed to the formation of only four possible gametes, i.e. M/St, m/T, M/m and T/St (Fig. 6), which are associated with alternate and adjacent-1 meiotic segregation modes, and suggest that there is no adjacent-2 segregation from these translocations in this species.

**Figure 7:**
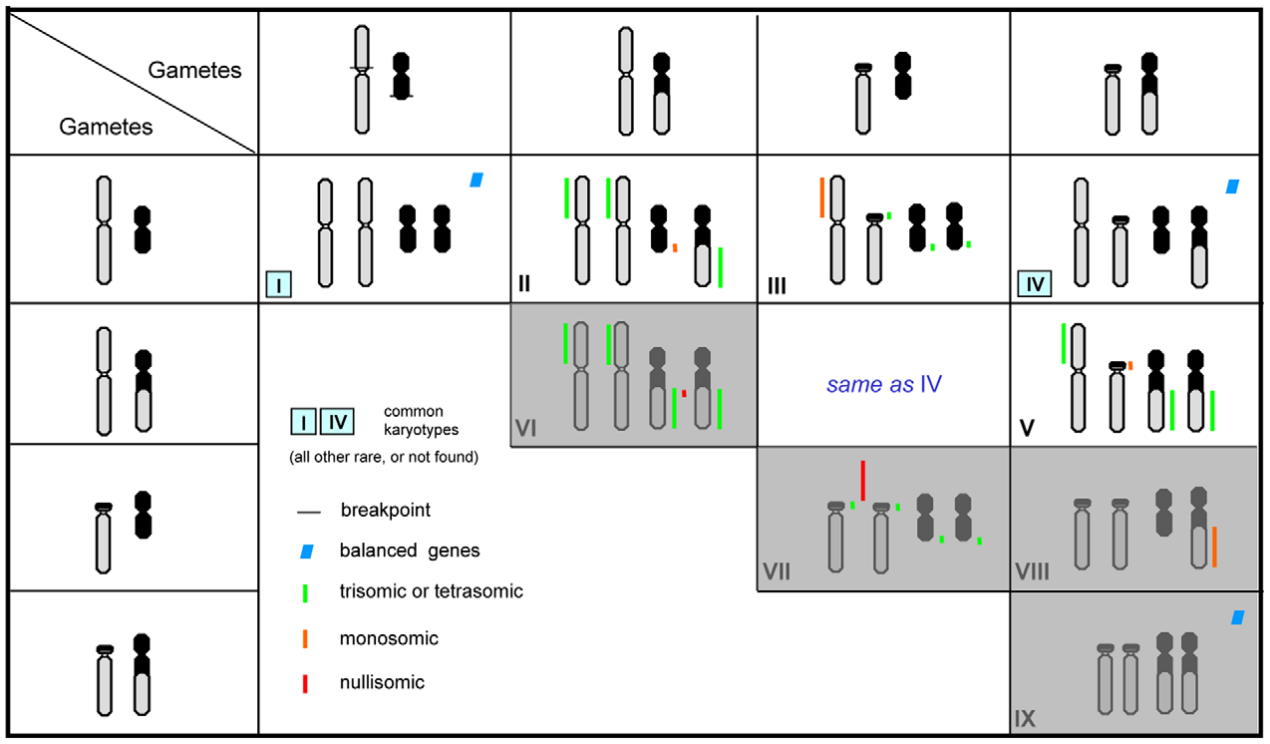
Diagrammatic representation of the nine expected karyomorphs resulting from the combination of four gametes produced by alternate and adjacent-1 segregations. Progenies with karyotypes in the grey box are absent (type VI, VII, VIII, IX) while those in white box are viable (type I, II, III, IV, V). For both gametes and progenies, only no. 1 and no. 6 chromosomes are given.

The possible explanation for higher frequencies of type I (81.8%) and IV (16.3%) karyomorphs is that alternate segregation predominates over adjacent-1 segregation. Alternate segregation can produce genetically normal (M/m) and complementary (T/St) gametes, which are genetically balanced without any duplications or deficiencies. When these gametes fuse with each other, three karyomorphic progenies would be expected: normal individuals (type I, MM/mm); individuals that are translocation heterozygotes (type IV, MT/mSt); and individuals that are translocation homozygotes (type IX, TT/StSt) (Figs. 6, 7). This is in accordance with our observations in most populations of *Quasipaa boulengeri* although translocation homozygotes (type IX) were not found in any population (Tables 1, 3). Similar cases with high alternate frequencies have been previously found in plants and animals, such as *Clarkia speciosa* [20], *Isotoma petraea* [21], Rye grass [22], [23], *Kalotermes approximates* [24] and *Periplaneta americana* [25], and especially in the genus *Oenothera* where segregation is always in the alternate mode [19], [26].

The frequency of the adjacent-1 segregation mode was found to be extremely low in this species. Karyotype II (MM/mSt), III (MT/mm), and V (MT/StSt), involving gametes M/St and m/T yielded by adjacent-1 mode, were found in few individuals in few populations (Tables 1, 3). Theoretically, the gametes derived from the adjacent-1 mode are genetically unbalanced for duplications or deficiencies, which could lead to lethality and infertility. This is, however, not always true. Occurrences of adjacent-1 mode have been found in plants and in invertebrates. It appears at a low frequency in *Hordeum vulgate*, and *Cochliomyia hominivorax* [27], [28]. This mode may even predominate as observed in *Allium atropurpureum, A. consanguinium, Chorthippus brunneus, Gomphocerus sibiricus* [29]–[32]. If gametes from both the adjacent-1 and alternate segregation fuse with each other in *Quasipaa boulengeri* translocation heterozygotes, seven karyomorphs associated with adjacent-1 mode should theoretically be observed including type II (MM/mSt), type III (MT/mm), type IV (MT/mSt), type V (MT/StSt), type VI (MM/StSt), type VII (TT/mm) and type VIII (TT/mSt) (Figs. 6, 7). Only three (Type II, III, and V) were found, which suggests that the adjacent-1 mode is uncommon or genetically detrimental in *Q. boulengeri*. Adjacent-2 segregation must not be viable in *Q. boulengeri*. Gametes resulting from the adjacent-2 mode would possess more duplications and/or deficiencies of chromosome segments. Some authors advocate that the adjacent disjunction that results in the movement of homologous centromeres to the same pole is extremely infrequent [31], [33], [34]. This may indicate that adjacent-2 orientations rarely occur during the meiosis. It should be also noticed that the meiotic pairing between no. 6 and no. 1 may often fail because of the very short pairing segments(Fig. 6)which can frequently convert the quadrivalent to a chain (rather than a ring) and make adjacent-2 segregations very unlikely [35].

### Absence of anticipated karyotypes

**Figure 8:**
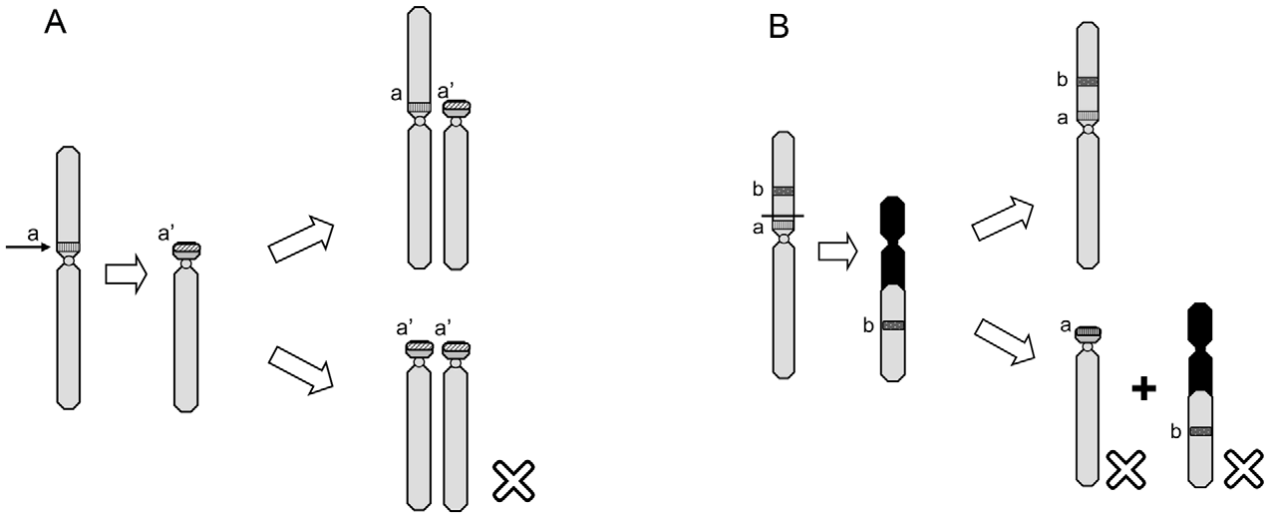
Diagram illustrates the possible mechanisms for parts of the absent karyomorphs. A. “recessive lethal mutation” mechanism. If one gene “a” locates at the translocation breakpoint (arrow indicated) and when the translocation occurs, “a” mutates to a recessive lethal gene “a' ”, and the progenies with genotype “a'a' ” will die while with “aa' ” will be viable. B. “position effect” mechanism. The breakpoint locates between two genes “a” and “b” on chromosome 1. When the translocation occurs, “b” will translocate to chromosome 6 and the relative position of “a” and “b” will be changed, then neither “a” nor “b” can function effectively and give rise to the death of progenies with either separated gene “a” or “b”. Once a progeny obtain a chromosome with normal gene order of “a” and “b”, it will be viable.

Theoretically, the union of the four occurring gametes (M/St, m/T, M/m and T/St) produced by alternate and adjacent-1 segregation can form nine kinds of karyologically different progeny, i.e. type I (MM/mm), II (MM/mSt), III (MT/mm), IV (MT/mSt), V (MT/StSt), VI (MM/StSt), VII (TT/mm), VIII (TT/mSt) and IX (TT/StSt) (Figs. 6, 7). Four anticipated karyotypes (VI, VII, VIII, and IX) were not found in any population. The absence of type IX is particularly confusing. Type IX (i.e. translocation homozygotes), would result from the fusion of translocated gametes (T/St), are genetically balanced without any duplication or deficiency, and can theoretically survive. Verified cases of homozygously fixed reciprocal translocations have been reported to occur in both plants and animals, such as Lewis rats [36], mice [37], different species of *Secale* [38], [39] and species in the genus *Datura* [40]. But, translocation homozygotes with lethal effects have also been observed in *Caenorhabditis elegans* [41], [42], and some species of *Drosophila* [43]–[45]. Some authors predicted that lethality of the translocation homozygosity (induced following “X-rays”) found in *Drosophila* may have one of two causes: recessive lethal mutations at the points where chromosomes broke and the position effect of the rearrangement [43], [45]. These two assumptions may explain the absence of type IX, together with type VII and VIII in *Quasipaa boulengeri* (Fig. 8A, B). In addition, it should be also noticed that either the occurring of adjacent-segregation can reduce the chances of segregating a balanced translocation into a gamete, or the translocation homozygotes were not found, just by chance. The absence of type VI, together with the rarity of types II, III, and V are possiblely caused by the genetic duplication or deficiency (Fig. 7). These two mechanisms can cause the reduction of the viability of the individuals and this is probably why only a very few or totally no frogs with these karyomorphs were found.

### Origin of chromosomal polymorphisms

**Figure 9:**
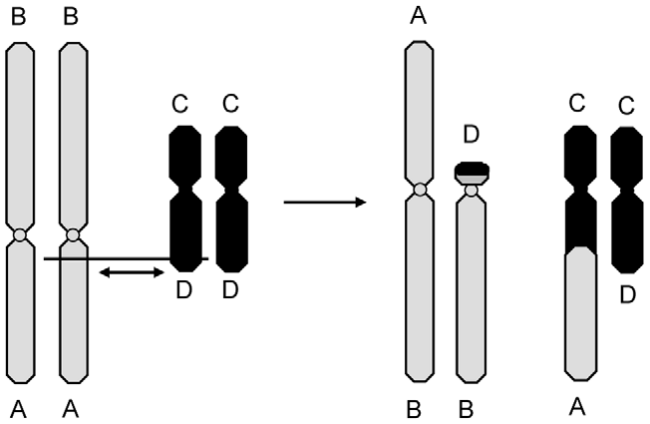
Diagrammatic representation of a reciprocal translocation occurring between one member of the chromosome pair 1 (AB) and pair 6 (CD) giving rise to two new chromosomes (BD and AC).

Why do translocation polymorphisms tend to be common in the species *Quasipaa boulengeri*? Interchanges are rare in nature in the heterozygous condition, and most translocations that occur in vertebrates are generally observed in single individuals [1], [3]. However in present study, there is a high frequency of the translocation heterozygotes (type IV) within and between different populations of *Q. boulengeri*. The reciprocal translocations can probably become evolutionarily fixed only when the interchanged regions are so minute that genetic unbalance involved in aneuploidy is not deleterious or the chromosomes regularly undergo alternate segregation. The latter is only likely to be the case when the chromosomes have interchanged virtually entire chromosome arms and then have distally localized chiasmata where it can be terminalized without difficulties [1]. Such as in the black flies, i.e. the genus *Prosimulium* and *Twinnia*, and midges, i.e. the genus *Chironomus*, whole-arm translocation have been evolutionarily fixed in various species [46]. Sometimes, similar translocation polymorphisms do become fixed in a species, such as the bird, *Megalaima zeylanica caniceps*. In that species, a translocation occured involving the exchange of a large segment of chromosome no. 1 and a small terminal region of a microchromosome and a chromosome chain formed during meiosis. In a limited sample of 11 individuals, five normal individuals (2♂, 3♀), five translocation heterozygotes (3♂, 2♀) and one translocation homozygote (1♀) were found [16]. Obviously, these three karyomorphs resulted from the union of gametes produced by alternate segregation and this segregation type may dominant in this bird. Similarly, in *Q. boulengeri*, the exchange involved almost the whole short arm of chromosome 1 and a very small chromosome segment from the long arm of chromosome 6 which is very similar to that of *Megalaima zeylanica caniceps* (Fig. 9). It is possible that a chain quadrivalent form and alternate segregation dominate in *Q. boulenger* which would result in the high frequencies of type IV such as the populations from Xuankouzhen, Hemingxiang-2 and Gaotangsi (Table 3). The whole-arm translocation probably resulted in the maintenance of the translocation polymorphisms in a few populations of *Q. boulengeri*.

**Figure 10:**
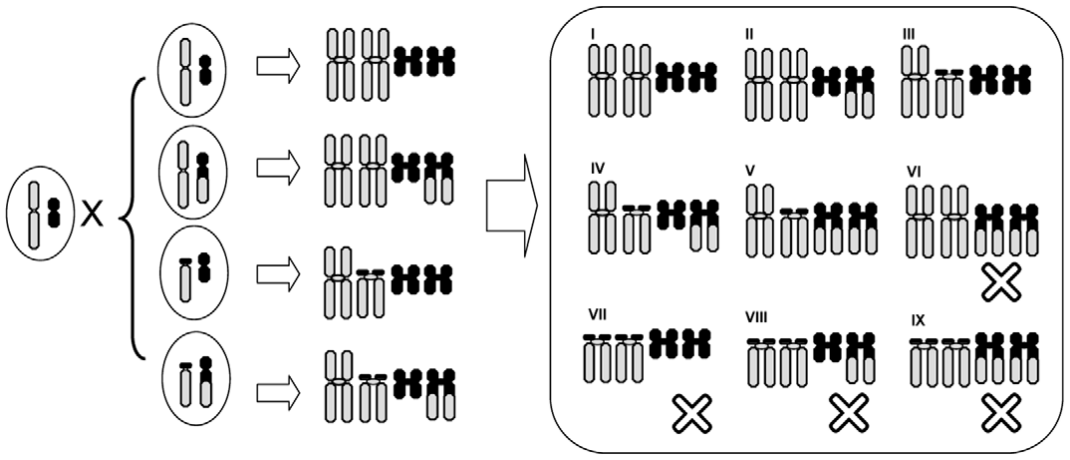
Diagrammatic representation of the possible dispersal of an individual with karyotype IV to the normal population. It can give rise to type I, II, III, and IV in F1. Only five karyotypes (I, II, III, IV and V) can be observed after F2, because the other four types (VI, VII, VIII and IX) are pridicted to die.

How does the translocation disperse among natural populations in *Quasipaa boulengeri*? The phylogenetic inference proved that all populations of *Q. boulengeri* grouped into a monophyletic clade with high support value, sister to *Q. spinosa* (COI+rRNA) or a clade containing *Q. spinosa* and *Q. cf. boulengeri* (rRNA+COI). In fact, the populations possessing translocated karyomorphs and those without any heteromorphic karyotypes did not diverge significantly from each other in DNA sequences sampled (Figs. 5A, B). We believe that the mutual translocation independently evolved just once in this species. The first step is possibly that a translocation rearrangement between chromosome no.1 and no.6 randomly occured in a single individual, then this translocation heterozygote (type IV, MT/mSt) individual mated with a normal one (type I, MM/mm) and produced more translocation heterozygote animals in the population. When the translocation heterozygotes mate with each other, all five karyomorphs can be produced in the same population (Fig. 10). Theoretically, an individual possessing each of karyotypes can disperse to a different population. Supposing an individual of type II (MM/mSt) moves into another population with type I individuals (normal), obtaining possible gametes of M/m and M/St during meiosis. Only hybrid offspring of type I and II will be produced after several generations. Similarly, if a type III migrates, two types of I and III will be produced in the future population. When each of these individuals disperses, the populations with two fixed types are anticipated to appear in the species. But this is not true in our data, which might suggest that the frequencies of both two types are so low that the opportunity for the dispersal of these types would be severely reduced. Significantly, if an individual with type IV (MT/mSt) or type V (MT/StSt) spreads, and mates with normal individual with gametes of M/m (type I), type IV must yield in F1 progenies (Fig. 10). Further, whenever type IV shows up in the population, the occurrences of all the five types of I, II, III, IV, and V are expected except VI, VII, VIII, and IX (death) in next progenies. Thus, it seems that the dispersal of each of both type IV and V is probably the mechanism for the translocation variations among populations. Virtually, for far lower frequency of the type V, the more reasonable explanation is that just the type IV is dispersing in various populations in the species.

## Materials and Methods

### Animals

Four hundred and seventy two adult frogs from 33 populations were used for karyological investigation in present study. Frogs were collected from 2006 to 2011 and consisted of 173 males and 298 females. The sampling localities, numbers of frogs and sexual ratios are provided in Table 1 and Fig. 1. All the specimens were obtained in the breeding season so that male specimens could be used to confirm identity of the species. Males and females were distinguished from each other both by morphology and by dissection to observe gonads. All animal work in this paper has been conducted according to relevant national and international guidelines. All animal care and experimental procedures were approved by the Chengdu Institute of Biology Animal Care and Use Committee.

### Mitotic Chromosome preparation

Mitotic metaphases were prepared from bone marrow technique described by Schmid et al. (2010) [5]. Chromosome preparations were examined using a Leica DMRA2 microscope.

Individuals with karyotype I (6♂, 4♀ from five populations) and karyotype IV (5♂, 5♀ from four populations) were chosen to measure the chromosome relative length and arm ratio, respectively (Tables 2, 5). The chromosomes were described following the nomenclature for centromeric position on chromosomes defined by Leven et al. (1964) [47]. Idiograms were also made using these measurements (Fig. 2). T tests were performed between the sum of the length of the four heteromorphic chromosomes from chromosome no. 1 and no. 6 designed as Z_1_+W_1_+Z_2_+W_2_ by Wang (2006) [14] and the sum of the length of the two normal homologues designed as (Z_1_+Z_2_)×2 (Table 4). Other slides were stored at room temperature for 2–4 days, dehydrated in ethanol series(70%, 90%, 100%, 3 min each)and stored at −20 C until used for fluorescence in situ hybridization.

**Table 5:** Quantitative data on the karyotype I and IV.

Type I				Type IV			
No.	RL(M±SD)	AR(M±SD)	C	No.	AR(M±SD)	RL(M±SD)	C
1	16.61±0.52	1.23±0.12	M	1−1	13.03±0.55	1.19±0.09	M
				1−2	8.32±0.60	7.11±0.11	T
2	13.92±0.75	2.51±0.41	Sm	2	11.34±0.42	2.49±0.21	Sm
3	11.5±0.87	2.31±0.19	Sm	3	9.63±0.70	2.54±0.54	Sm
4	10.77±0.45	1.52±0.18	M	4	8.96±0.39	1.47±0.13	M
5	9.85±0.94	1.35±0.12	M	5	8.19±0.57	1.32±0.12	M
6	6.09±0.34	1.27±0.11	m	6−1	5.01±0.34	1.17±0.11	m
				6−2	9.02±0.66	3.35±0.31	St
7	5.38±0.24	2.37±0.41	sm	7	4.69±0.20	2.04±0.30	sm
8	5.25±0.17	1.38±0.18	m	8	4.38±0.28	1.25±0.14	m
9	4.89±0.41	2.11±0.3	sm	9	4.08±0.21	2.00±0.24	sm
10	4.64±0.31	1.28±0.2	m	10	3.92±0.23	1.22±0.08	m
11	4.12±0.28	1.18±0.2	m	11	3.47±0.34	1.23±0.16	m
12	3.68±0.28	1.19±0.14	m	12	3.19±0.27	1.16±0.16	m
13	3.30±0.33	1.19±0.14	m	13	2.76±0.26	1.18±0.16	m

RL: relative length (one chromosome length/total chromosome length); AR: arm radio (long arm length/short arm length); C: Cenromere position;.

M =  large metacentric chromosome; m =  small metacentric chromosome; Sm =  large submetacentric chromosome; sm =  small submetacentric chromosome; St =  large subtelocentric chromosome; T =  large telocentric chromosome.1−1, 1−2: Two homologues of chromosome no. 1; 6−1, 6−2: Two homologues of chromosome no. 6.

### 5S rDNA probe construction and fluorescence in situ hybridization (FISH)

Genomic DNA was extracted from liver or muscle tissues by standard Proteinase K method [48]. 5S rDNA was amplified using forward 5′-GCCTACGGCCACACCAC-3′ and reverse 5′-AAGCCTACGACACCTGGTATTC-3′ primers. Probes were labeled by PCR with biotin-16-Dutp (Roche) or Digoxigenin-11-dUTP (Roche) following the procedure described by Bi et al. (2009) [49] with small modification. The PCR program included 3 min initial denaturation, 30 cycles of 94°C/50°C/72°C for 30 s/30 s/1 min followed with a 10 min extension at 72°C.

Mitotic chromosomes were used for 5S rDNA FISH analysis following the procedure reported by Zhang et al (2007) [50] and Bi et al., (2009) [49] with a few modifications. The hybridization mix contained 10 ng/µl probe, 10% dextran sulphate, 0.1% SDS, 1X denhardt, 50% deionized formamide in 2× SSC, 83°C for 7 min and cooled down immediately by putting on ice for at least 10 min. Biotinylated or Digoxigenated 5S probes were detected with fluorescein-labeled avidin DCS (Vector) or anti-digoxigenin-fluorescin fab fragments (Roche) at 37°C for about 1 h. After hybridization, chromosomes were counterstained with propidium iodide (PI) in antifade solution (Vector) at 37°C for approximately 45 min. Hybridization signals were detected using a Leica DMRA2 fluorescent microscope equipped with appropriate filter sets for FITC and PI. Images were captured using a Leica DFC490 CCD camera with Qwin V3 and Qgo software.

### Phylogenetic analysis

A total of 133 specimens were sampled for molecular phylogenetic analysis, including 116 *Quasipaa boulengeri* individuals from 20 collecting sites and 17 individuals belonging to 13 species closely related to *Q. boulengeri*. Three fragments from the mitochondrial genome were selected for sequencing (Table S1). The first fragment is part of the COI gene, and was 628 bp in length. The other two fragments are part of the 12S and part of the 16S genes, and were approximately 362 bp and 534 bp before alignment, respecvely. The COI fragment of 96 specimens and both the 12S and 16S fragments of 52 specimens were sequenced in this study. Other sequences were obtained from previous studies [15], [51], [52]. Details of the sampling and polymerase chain reaction primers are presented in Tables S1 and S2, respectively. Sequence alignment was conducted with ClustalX version 1.83 [53] and checked by eye. The amino acid sequences for coding regions and the rRNA secondary structures of *Xenopus laevis* [54] were used for checking.

For phylogenetic reconstruction, the three fragments were combined, and two data sets were analyzed separately. The rRNA+COI data set covered a relatively wider phylogenetic range, and all haplotypes in this data set contained the 12S and 16S sequences. The COI+rRNA data set was a more comprehensive sampling of *Quasipaa boulengeri*, especially the populations found in Western Sichuan Basin which possessed both heteromorphic and homomorphic karyotypes. All haplotypes in this data set contained the COI fragment sequence. Three and one species were selected as outgroups for the rRNA+COI and COI+rRNA data sets based on the current understanding of their phylogenetic relationships, respectively [15]. A three-partition strategy was applied to partition both data sets. It defined each of the 12S, 16S, and COI genes as a separate partition. Both maximum likelihood (ML) and Bayesian approaches were conducted on these data sets. The Bayesian information criterion (BIC) and corrected Akaike information criterion (AICc) implemented in jModelTest version 0.1.1 [55] were used to select and evolutionary model that best fit each data partition [56], [57]. Models selected are provided in Table S3.

The ML analysis was conducted using RAxML version 7.2.6 [58]. This program applies one substitution model (GTR+G or GTR+I+G) to all DNA data partitions. As the selected models are relatively simple (Table S3), the GTR+G model was used. The rapid hill-climbing algorithm [59] was used and 200 inferences were executed. To estimate nodal support, nonparametric bootstrap proportions [60] with 1000 replicates were used. The Bayesian analysis was conducted using MrBayes version 3.1.2 [61]. For the rRNA+COI data set, a same set of model parameters were chosen by both BIC and AICc. While for the COI+rRNA data, different sets of parameters were chosen by the two criterions. Consequently, the Bayesian analysis was conducted on the COI+rRNA data with parameters chosen by BIC or AICc, separately, and the results were compared [57]. Four Markov chains were used and the data was run for 5 million generations to allow adequate time for convergence. Trees were sampled every 500 generations and the last 5000 sample trees were used to estimate the consensus tree and the Bayesian posterior probabilities.

## Supporting Information

Table S1 Sampling information for molecular phylogenetic analysis. For some individuals, not all the three fragments were sequenced, causing one sequence be categorized into more than one haplotype. * All rRNA+COI haplotypes contained the 12S and 16S sequences, and all COI+rRNA haplotypes contained the COI sequence.
(DOC)

Table S2 Primers used in PCR and sequencing of *Quasipaa boulengeri* in this study.
(DOC)

Table S3 Models selected for data partitions by the Bayesian information criterion (BIC) and corrected Akaike information criterion (AICc) * All rRNA+COI haplotypes contained the 12S and 16S sequences, and all COI+rRNA haplotypes contained the COI sequence.
(DOC)

## References

[1] White MJD (1973) Animal cytology and evolution London: Cambridge University Press. 933 p.

[2] Futuyma D (1997) Evolutionary Biology. Sinauer Associates: Sunderland, Massachusetts. 763 p.

[3] Chiarelli A, Capanna E (1973) Cytotaxonomy and vertebrate evolution. London. New York: Academic Press. 783 p.

[4] Schmid M, Steinlein C, Haaf T (2004) Chromosome banding in Amphibia XXX. Karyotype aberrations in cultured fibroblast cells. Cytogenetic and Genome Research 104: 277–282. 15162051 10.1159/000077502

[5] Schmid M, Steinlein C, Bogart JP, Feichtinger W, León P, et al (2010) The chromosomes of terraranan frogs. Insights into vertebrate cytogenetics. Cytogenetic and Genome Research 130–131: 1–568. 10.1159/000301339 21063086

[6] Siqueira-Jr S, Ananias F, Recco-Pimentel S (2004) Cytogenetics of three Brazilian species of Eleutherodactylus (Anura, Leptodactylidae) with 22 chromosomes and re-analysis of multiple translocations in *E. binotatus* . Genetics and Molecular Biology 27: 363–372.

[7] Fei L, Hu SQ, Ye CY, Huang YZ (2009) Fauna Sinica. Amphibia Vol. 3 Anura Ranidae. Beijing: Science Press. 957 p.

[8] Chen WY, Wang ZS, Wang XZ, Yang YH, Sun QL (1983) A Comparative Study of the Karyotypes From six Species of Frogs in Sichuan. Zoological Research 4: 83–88.

[9] Wang ZS, Wang XZ, Chen WY (1983) A comparative study on constitutive heterochromatin and mucleolus-organization regions (NORs) of three species of the genus *Rana* . Acta Herpetologica Sinica 2: 1–6.

[10] Li SS, Hu JS (1999) The karyotype evolution and infraspecies variation of geographical population of anura genus of *Paa* from China. China Zoological Society Zoological Studies in China. Beijing: Chinese Forestry Publishing House. 976–982.

[11] Li SS, Hu JS (1996) The study on the karyotypes, C-banding and Ag-NORs of four *Paa* Species in China. Zoological Research 17: 84–88.

[12] Zhang JY, Gu XM (1997) A study on karyotype and Ag-NORs of *Paa boulengeri* from Shuicheng of Guizhou. Journal Guizhou Normal University (Natural Science) 15: 48–51.

[13] Hu JS (2004) Molecular phylogenetic studies on Spinosae group (genus *Paa*) in China (Amphibian, Anura, Ranidae). Kunming: Yunnan University. 97 p.

[14] Wang D (2006) Chromosome Research on *Paa bouelngeri* and *Paa yunnanensis* (Ranidae: *Paa*). Chengdu: Sichuan University. 48 p.

[15] Che J, Hu J, Zhou W, Murphy RW, Papenfuss TJ, et al (2009) Phylogeny of the Asian spiny frog tribe Paini (Family Dicroglossidae) sensu Dubois. Molecular Phylogenetics and Evolution 50: 59–73. 18992827 10.1016/j.ympev.2008.10.007

[16] Kaul D, Ansari H (1981) Chromosomal polymorphism in a natural population of the northern green barbet, *Megalaima zeylanica caniceps* (Franklin)(Piciformes: Aves). Genetica 54: 241–245.

[17] Nai WH, Liu RQ, Chen YZ, Wang JH (1999) A Study of Chromosome Translocation of Francois' Monkey by Fluoresence in situ Hybridization (FISH). Hereditas (Beijing) 21: 1–3.

[18] Kingston HM (2002) ABC of Clinical Genetics. London: BMJ Books. 120 p.

[19] Hartl DL, Jones EW (1998) Genetics: principles and analysis. Sudbury, Massachusetts: Jones and Bartlett Publishers. 1367 p.

[20] Bloom WL (1974) Origin of reciprocal translocations and their effect in Clarkia speciosa. Chromosoma 49: 61–76.

[21] James S (1970) Complex hybridity in Isotoma petraea. II. Components and operation of a possible evolutionary mechanism. Heredity 25: 53–78.

[22] Lawrence C (1963) The orientation of multiple associations resulting from interchange heterozygosity. Genetics 48: 347–350. 17248155 10.1093/genetics/48.3.347 PMC1210475

[23] Sun S, Rees H (1967) Genotypic control of chromosome behaviour in rye. IX. The effect of selection on the disjunction frequency of interchange associations. Heredity 22: 249–254.

[24] Syren RM, Luykx P (1981) Geographic variation of sex-linked translocation heterozygosity in the termite *Kalotermes approximatus* Snyder (Insecta: Isoptera). Chromosoma 82: 65–88.

[25] Lewis K, John B (1957) Studies on *Periplaneta americana*. II. Interchange heterozygosity in isolated populations. Heredity 11: 11–22.

[26] Cleland RE (1926) Cytological studies of meiosis in anthers of *Oenothera muricata* Botanical Gazette. 82: 55–70.

[27] Smith L (1941) An inversion, a reciprocal translocation, trisomics, and tetraploids in barley. J Agric Res 63: 741–750.

[28] LaChance LE, Riemann JG, Hopkins D (1964) A reciprocal translocation in *Cochliomyia hominivorax* (Diptera: Calliphoridae). Genetic and cytological evidence for preferential segregation in males. Genetics 49: 959–972. 14181488 10.1093/genetics/49.6.959 PMC1210628

[29] John B, Hewitt G (1963) A spontaneous interchange in *Chorthippus brunneus* with extensive chiasma formation in an interstitial segment. Chromosoma 14: 638–650.

[30] Koul A (1966) Structural hybridity in *Allium atropupureum* Waldst and Kit. J Cytol Genet 1: 87–89.

[31] Gohil R, Koul A (1978) Structural hybridity in *Allium consanguineum* . Cytologia 43: 243–247.

[32] Gosálvez J, López-Fernández C, Garcia-Lafuente R (1982) A spontaneous translocation heterozygote involving centromere regions in *Gomphocerus sibiricus* (L.)(Orthoptera: Acrididae). Chromosoma 86: 49–57.

[33] Shalev A, Ladizinsky G (1976) The segregation pattern of a translocation quadrivalent. Chromosoma 57: 297–308.

[34] Pierce BA (2002) Genetics: A conceptual approach. San Francisco WH Freeman. 736 p.

[35] Rickards GK (1983) Orientation behavior of chromosome multiples of interchange (reciprocal translocation) heterozygotes. Annu Rev Genet 17: 443–498. 6364963 10.1146/annurev.ge.17.120183.002303

[36] Yosida TH (1980) Cytogenetics of the black rat, karyotype evolution and species differentiation. Tokyo: University of Tokyo Press. 235 p.

[37] Cartes T, Lyon MF, Phillips RJS (1955) Gene-tagged chromosome translocations in eleven stocks of mice. J Genet 53: 154–166.

[38] Darlington CD (1937) Recent advances in cytology. London: Churchill. 671 p.

[39] Price S (1955) Irradiation and interspecific hybridization in Secale. Genetics 40: 651–667. 17247578 10.1093/genetics/40.5.651 PMC1209747

[40] Blakeslee AF (1927) Genetics of *Datura*. Verh. 5. Intern. Kongr. Vererb. wiss. Berlin, 1927. Z. indukt. Abstamm.- u. Vererb-L Suppl. 117–130

[41] Fodor A, Deak P (1985) The isolation and genetic analysis of a *Caenorhabditis elegants* translocation (szT1) strain bearing an X-chromosome balancer. Journal of Genetics 64: 143–157.

[42] McKim KS, Howell AM, Rose AM (1988) The effects of translocations on recombination frequency in *Caenorhabditis elegans* . Genetics 120: 987–1001. 3224815 10.1093/genetics/120.4.987 PMC1203590

[43] Muller H, Altenburg E (1930) The frequency of translocations produced by X-rays in *Drosophila* . Genetics 15: 283–311. 17246601 10.1093/genetics/15.4.283 PMC1201066

[44] Kaufman TC (1978) Cytogenetic analysis of chromosome 3 in *Drosophila melanogaster*: isolation and characterization of four new alleles of the proboscipedia (pb) locus. Genetics 90: 579–596. 17248871 10.1093/genetics/90.3.579 PMC1213906

[45] Roberts PA (1976) The genetics of chromosome Aberration. In: Ashburner M, Novitski E, editors. The genetics and biology of *Drosophila*. New York and London: Academic Press. 68–184.

[46] Lande R (1979) Effective deme sizes during long-term evolution estimated from rates of chromosomal rearrangement. Evolution 33: 234–251. 28568063 10.1111/j.1558-5646.1979.tb04678.x

[47] Levan A, Fredga K, Sandberg AA (1964) Nomenclature for centromeric position on chromosomes. Hereditas 52: 201–220.

[48] Sambrook J, Russell D (2001) Molecular Cloning: A Laboratory Manual. Cold Spring Harbor, New York: Cold Spring Harbor Press.

[49] Bi K, Bogart JP, Fu J (2009) A populational survey of 45S rDNA polymorphism in the Jefferson salamander *Ambystoma jeffersonianum* revealed by fluorescence in situ hybridization (FISH) Current Zoology. 55: 145–149.

[50] Zhang LL, Bao ZM, Wang S, Huang X, Hu JJ (2007) Chromosome rearrangements in Pectinidae (Bivalvia: Pteriomorphia) implied based on chromosomal localization of histone H3 gene in four scallops. Genetica 130: 193–198. 16909332 10.1007/s10709-006-9006-8

[51] Che J, Chen HM, Yang JX, Jin JQ, Jiang K, et al (2012) Universal COI primers for DNA barcoding amphibians. Molecular Ecology Resources 12: 247–258. 22145866 10.1111/j.1755-0998.2011.03090.x

[52] Zhou Y, Zhang JY, Zheng RQ, Yu BG, Yang G (2009) Complete nucleotide sequence and gene organization of the mitochondrial genome of *Paa spinosa* (Anura: Ranoidae). Gene 447: 86–96. 19631263 10.1016/j.gene.2009.07.009

[53] Thompson JD, Gibson TJ, Plewniak F, Jeanmougin F, Higgins DG (1997) The Clustal X windows interface: flexible strategies for multiple sequence alignment aided by quality analysis tools. Nucleic Acids Research 25: 4876–4882. 9396791 10.1093/nar/25.24.4876 PMC147148

[54] Cannone J, Subramanian S, Schnare M, Collett J, D'Souza L, et al (2002) The comparative RNA web (CRW) site: an online database of comparative sequence and structure information for ribosomal, intron, and other RNAs. BMC bioinformatics 3: 2. 11869452 10.1186/1471-2105-3-2 PMC65690

[55] Posada D (2008) jModelTest: phylogenetic model averaging. Molecular biology and evolution 25: 1253–1256. 18397919 10.1093/molbev/msn083

[56] Posada D, Buckley TR (2004) Model selection and model averaging in phylogenetics: advantages of Akaike information criterion and Bayesian approaches over likelihood ratio tests. Systematic Biology 53: 793–808. 15545256 10.1080/10635150490522304

[57] Tamura K, Peterson D, Peterson N, Stecher G, Nei M, et al (2011) MEGA5: molecular evolutionary genetics analysis using maximum likelihood, evolutionary distance, and maximum parsimony methods. Molecular biology and evolution 28: 2731–2739. 21546353 10.1093/molbev/msr121 PMC3203626

[58] Stamatakis A (2006) RAxML-VI-HPC: maximum likelihood-based phylogenetic analyses with thousands of taxa and mixed models. Bioinformatics 22: 2688–2690. 16928733 10.1093/bioinformatics/btl446

[59] Stamatakis A, Blagojevic F, Nikolopoulos DS, Antonopoulos CD (2007) Exploring new search algorithms and hardware for phylogenetics: RAxML meets the IBM cell. The Journal of VLSI Signal Processing 48: 271–286.

[60] Felsenstein J (1985) Confidence limits on phylogenies: an approach using the bootstrap. Evolution: 783–791. 10.1111/j.1558-5646.1985.tb00420.x 28561359

[61] Ronquist F, Huelsenbeck JP (2003) MrBayes 3: Bayesian phylogenetic inference under mixed models. Bioinformatics 19: 1572–1574. 12912839 10.1093/bioinformatics/btg180

