# Supplementary material for: A De Novo Case of Floating Chromosomal Polymorphisms by Translocation in Quasipaa boulengeri (Anura, Dicroglossidae)
Source: PLoS One. 2012 Oct 3;7(10):e46163. doi: 10.1371/journal.pone.0046163 (PMC3463521; doi:10.1371/journal.pone.0046163)
Supplement: Table S1 — Sampling information for molecular phylogenetic analysis. For some individuals, not all the three fragments were sequenced, causing one sequence be categorized into more than one haplotype. * All rRNA+COI haplotypes contained the 12S and 16S sequences, and all COI+rRNA haplotypes contained the COI sequence. (DOC) [file pone.0046163.s001.doc]

| Table S1 Sampling information for molecular phylogenetic analysis. For some individuals, not all the three fragments were sequenced, causing one sequence be categorized into more than one haplotype. | | | | | | | | |
| --- | --- | --- | --- | --- | --- | --- | --- | --- |
| Species | Localities | Voucher specimens | Haplotype* | | GenBank No. mtDNA | | | References |
|  |  |  | rRNA+COI | COI+rRNA | 12S | 16S | COI |  |
| *Quasipaa boulengeri* | Hongkouxiang, Dujiangyan City, Sichuan, China (1) | XM2028 | H14/H15/H17 | — | JX629496 | JX629536 |  | This study |
| *Q. boulengeri* | Hongkouxiang, Dujiangyan City, Sichuan, China (1) | XM2031 | H15 | B11 | JX629508 | JX629535 | JX629604 | This study |
| *Q. boulengeri* | Hongkouxiang, Dujiangyan City, Sichuan, China (1) | XM2029 | — | B8/B9/B10/B12 | — | — | JX629605 | This study |
| *Q. boulengeri* | Hongkouxiang, Dujiangyan City, Sichuan, China (1) | XM2037 | — | B11 | — | — | JX629606 | This study |
| *Q. boulengeri* | Hongkouxiang, Dujiangyan City, Sichuan, China (1) | XM2038 | — | B8/B9/B10/B12 | — | — | JX629607 | This study |
| *Q. boulengeri* | Hongkouxiang, Dujiangyan City, Sichuan, China (1) | XM2039 | — | B8/B9/B10/B12 | — | — | JX629608 | This study |
| *Q. boulengeri* | Hongkouxiang, Dujiangyan City, Sichuan, China (1) | XM2946 | — | B8/B9/B10/B12 | — | — | JX629609 | This study |
| *Q. boulengeri* | Hongkouxiang, Dujiangyan City, Sichuan, China (1) | XM2961 | — | B11 | — | — | JX629610 | This study |
| *Q. boulengeri* | Mt. Qingcheng, Sichuan, China (3) | XM3481 | H17 | B9 | JX629512 | JX629554 | JX629636 | This study |
| *Q. boulengeri* | Mt. Qingcheng, Sichuan, China (3) | XM3482 | H17 | B9 | JX629506 | JX629555 | JX629637 | This study |
| *Q. boulengeri* | Mt. Qingcheng, Sichuan, China (3) | XM3076 | — | B8/B9/B10/B12 | — | — | JX629638 | This study |
| *Q. boulengeri* | Mt. Qingcheng, Sichuan, China (3) | XM3480 | — | B9 | — | — | JX629639 | This study |
| *Q. boulengeri* | Mt. Qingcheng, Sichuan, China (3) | XM3483 | — | B9 | — | — | JX629640 | This study |
| *Q. boulengeri* | Mt. Qingcheng, Sichuan, China (3) | XM3484 | — | B9 | — | — | JX629641 | This study |
| *Q. boulengeri* | Mt. Qingcheng, Sichuan, China (3) | XM3485 | — | B9 | — | — | JX629642 | This study |
| *Q. boulengeri* | Mt. Qingcheng, Sichuan, China (3) | XM2926 | — | B9 | — | — | JX629643 | This study |
| *Q. boulengeri* | Mt. Qingcheng, Sichuan, China (3) | XM2928 | — | B9 | — | — | JX629644 | This study |
| *Q. boulengeri* | Xilingzhen, Dayi Co., Sichuan, China (5) | XM3528 | H14 | B7 | JX629490 | JX629570 | JX629660 | This study |
| *Q. boulengeri* | Xilingzhen, Dayi Co., Sichuan, China (5) | XM3527 | H17 | B9 | JX629514 | JX629569 | JX629659 | This study |
| *Q. boulengeri* | Xilingzhen, Dayi Co., Sichuan, China (5) | XM3526 | — | B8/B9/B10/B12 | — | — | JX629655 | This study |
| *Q. boulengeri* | Xilingzhen, Dayi Co., Sichuan, China (5) | XM3512 | — | B8/B9/B10/B12 | — | — | JX629656 | This study |
| *Q. boulengeri* | Xilingzhen, Dayi Co., Sichuan, China (5) | XM3515 | — | B8/B9/B10/B12 | — | — | JX629657 | This study |
| *Q. boulengeri* | Xilingzhen, Dayi Co., Sichuan, China (5) | XM3516 | — | B8/B9/B10/B12 | — | — | JX629658 | This study |
| *Q. boulengeri* | Xilingzhen, Dayi Co., Sichuan, China (5) | XM3517 | — | B7 | — | — | JX629661 | This study |
| *Q. boulengeri* | Xilingzhen, Dayi Co., Sichuan, China (5) | XM3518 | — | B8/B9/B10/B12 | — | — | JX629662 | This study |
| *Q. boulengeri* | Xilingzhen, Dayi Co., Sichuan, China (5) | XM3525 | — | B8/B9/B10/B12 | — | — | JX629663 | This study |
| *Q. boulengeri* | Gaotangsi, Dayi Co., Sichuan, China (12) | XM3383 | H14/H15/H17 | — | JX629493 | JX629534 |  | This study |
| *Q. boulengeri* | Gaotangsi, Dayi Co., Sichuan, China (12) | XM3008 | H17 | B9 | JX629495 | JX629533 | JX629598 | This study |
| *Q. boulengeri* | Gaotangsi, Dayi Co., Sichuan, China (12) | XM3007 | — | B8/B9/B10/B12 | — | — | JX629599 | This study |
| *Q. boulengeri* | Gaotangsi, Dayi Co., Sichuan, China (12) | XM3028 | — | B8/B9/B10/B12 | — | — | JX629600 | This study |
| *Q. boulengeri* | Gaotangsi, Dayi Co., Sichuan, China (12) | XM3382 | — | B8/B9/B10/B12 | — | — | JX629601 | This study |
| *Q. boulengeri* | Gaotangsi, Dayi Co., Sichuan, China (12) | XM3384 | — | B8/B9/B10/B12 | — | — | JX629602 | This study |
| *Q. boulengeri* | Gaotangsi, Dayi Co., Sichuan, China (12) | XM3385 | — | B8/B9/B10/B12 | — | — | JX629603 | This study |
| *Q. boulengeri* | Pinglezhen, Qionglai Co., Sichuan, China (21) | XM3643 | H17 | B9 | JX629492 | JX629552 | JX629634 | This study |
| *Q. boulengeri* | Pinglezhen, Qionglai Co., Sichuan, China (21) | XM3644 | H17 | B9 | JX629500 | JX629553 | JX629635 | This study |
| *Q. boulengeri* | Mt. Tiantai-1, Qionglai Co., Sichuan, China (22) | XM3621 | H14/H15/H17 | — | JX629503 | JX629544 |  | This study |
| *Q. boulengeri* | Mt. Tiantai-1, Qionglai Co., Sichuan, China (22) | XM3590 | H15 | B11 | JX629504 | JX629546 | JX629613 | This study |
| *Q. boulengeri* | Mt. Tiantai-1, Qionglai Co., Sichuan, China (22) | XM3619 | H15 | B11 | JX629498 | JX629538 | JX629617 | This study |
| *Q. boulengeri* | Mt. Tiantai-1, Qionglai Co., Sichuan, China (22) | XM3623 | H15 | B11 | JX629489 | JX629541 | JX629618 | This study |
| *Q. boulengeri* | Mt. Tiantai-1, Qionglai Co., Sichuan, China (22) | QLY385 | H16 | — | JX629487 | JX629543 |  | This study |
| *Q. boulengeri* | Mt. Tiantai-1, Qionglai Co., Sichuan, China (22) | XM3625 | H16 | B10 | JX629486 | JX629537 | JX629619 | This study |
| *Q. boulengeri* | Mt. Tiantai-1, Qionglai Co., Sichuan, China (22) | XM3591 | H17 | B9 | JX629499 | JX629542 | JX629614 | This study |
| *Q. boulengeri* | Mt. Tiantai-1, Qionglai Co., Sichuan, China (22) | XM3603 | H17 | B9 | JX629494 | JX629540 | JX629615 | This study |
| *Q. boulengeri* | Mt. Tiantai-1, Qionglai Co., Sichuan, China (22) | XM3617 | H17 | B9 | JX629502 | JX629545 | JX629616 | This study |
| *Q. boulengeri* | Mt. Tiantai-1, Qionglai Co., Sichuan, China (22) | QLY392 | H17 | B9 | JX629507 | JX629539 | JX629620 | This study |
| *Q. boulengeri* | Mt. Tiantai-1, Qionglai Co., Sichuan, China (22) | QLY373 | — | B8/B9/B10/B12 | — | — | JX629611 | This study |
| *Q. boulengeri* | Mt. Tiantai-1, Qionglai Co., Sichuan, China (22) | QLY378 | — | B8/B9/B10/B12 | — | — | JX629612 | This study |
| *Q. boulengeri* | Mt. Tiantai-1, Qionglai Co., Sichuan, China (22) | QLY377 | — | B8/B9/B10/B12 | — | — | JX629654 | This study |
| *Q. boulengeri* | Mt. Tiantai-2, Qionglai Co., Sichuan, China (23) | XM3601 | H14/H15/H17 | — | JX629510 | JX629525 |  | This study |
| *Q. boulengeri* | Mt. Tiantai-2, Qionglai Co., Sichuan, China (23) | QLY389 | H14/H15/H17 | — | JX629517 | JX629527 |  | This study |
| *Q. boulengeri* | Mt. Tiantai-2, Qionglai Co., Sichuan, China (23) | QLY391 | H17 | B9 | JX629516 | JX629526 | JX629590 | This study |
| *Q. boulengeri* | Mt. Tiantai-2, Qionglai Co., Sichuan, China (23) | XM3598 | H17 | B9 | JX629515 | JX629530 | JX629586 | This study |
| *Q. boulengeri* | Mt. Tiantai-2, Qionglai Co., Sichuan, China (23) | XM3599 | H17 | B9 | JX629513 | JX629524 | JX629587 | This study |
| *Q. boulengeri* | Mt. Tiantai-2, Qionglai Co., Sichuan, China (23) | XM3600 | H17 | B9 | JX629511 | JX629529 | JX629588 | This study |
| *. boulengeri* | Mt. Tiantai-2, Qionglai Co., Sichuan, China (23) | XM3602 | H17 | B9 | JX629509 | JX629528 | JX629589 | This study |
| *Q. boulengeri* | Mt. Tiantai-2, Qionglai Co., Sichuan, China (23) | QLY390 | — | B8/B9/B10/B12 |  |  | JX629591 | This study |
| *Q. boulengeri* | Bifeng Valley, Yaan City, Sichuan, China (24) | XM3570 | H18 | B12 | JX629497 | JX629523 | JX629572 | This study |
| *Q. boulengeri* | Bifeng Valley, Yaan City, Sichuan, China (24) | XM3580 | H18 | B12 | JX629505 | JX629522 | JX629573 | This study |
| *Q. boulengeri* | Bifeng Valley, Yaan City, Sichuan, China (24) | XM3571 | — | B8/B9/B10/B12 | — | — | JX629574 | This study |
| *Q. boulengeri* | Bifeng Valley, Yaan City, Sichuan, China (24) | XM3573 | — | B8/B9/B10/B12 | — | — | JX629575 | This study |
| *Q. boulengeri* | Bifeng Valley, Yaan City, Sichuan, China (24) | XM3574 | — | B8/B9/B10/B12 | — | — | JX629576 | This study |
| *Q. boulengeri* | Bifeng Valley, Yaan City, Sichuan, China (24) | XM3575 | — | B8/B9/B10/B12 | — | — | JX629577 | This study |
| *Q. boulengeri* | Bifeng Valley, Yaan City, Sichuan, China (24) | XM3582 | — | B8/B9/B10/B12 | — | — | JX629578 | This study |
| *Q. boulengeri* | Bifeng Valley, Yaan City, Sichuan, China (24) | XM3583 | — | B8/B9/B10/B12 | — | — | JX629579 | This study |
| *Q. boulengeri* | Mt. Emei, Sichuan, China (26) | XM3080 | H14/H15/H17 | — | JX629501 | JX629531 |  | This study |
| *Q. boulengeri* | Mt. Emei, Sichuan, China (26) | XM3110 | H14/H15/H17 | — | JX629491 | JX629532 |  | This study |
| *Q. boulengeri* | Mt. Emei, Sichuan, China (26) | XM1934 | — | B8/B9/B10/B12 | — | — | JX629592 | This study |
| *Q. boulengeri* | Mt. Emei, Sichuan, China (26) | XM1971 | — | B8/B9/B10/B12 | — | — | JX629593 | This study |
| *Q. boulengeri* | Mt. Emei, Sichuan, China (26) | XM1979 | — | B8/B9/B10/B12 | — | — | JX629594 | This study |
| *Q. boulengeri* | Mt. Emei, Sichuan, China (26) | XM3062 | — | B11 | — | — | JX629595 | This study |
| *Q. boulengeri* | Mt. Emei, Sichuan, China (26) | XM3063 | — | B8/B9/B10/B12 | — | — | JX629596 | This study |
| *Q. boulengeri* | Mt. Emei, Sichuan, China (26) | XM3074 | — | B8/B9/B10/B12 | — | — | JX629597 | This study |
| *Q. boulengeri* | Xinglongzhen, Youyang Co., Chongqing, China (29) | XM3164 | H21 | B5 | JX629468 | JX629567 | JX629666 | This study |
| *Q. boulengeri* | Xinglongzhen, Youyang Co., Chongqing, China (29) | XM3176 | H22 | B4 | JX629470 | JX629568 | JX629664 | This study |
| *Q. boulengeri* | Xinglongzhen, Youyang Co., Chongqing, China (29) | XM3181 | H22 | B4 | JX629471 | JX629571 | JX629665 | This study |
| *Q. boulengeri* | Xinglongzhen, Youyang Co., Chongqing, China (29) | XM3173 | H23 | B3 | JX629472 | JX629566 | JX629667 | This study |
| *Q. boulengeri* | Kuankuoshui, Suiyang, Guizhou, China (30) | XM3392 | H24 | B21 | JX629519 | JX629547 | JX629621 | This study |
| *Q. boulengeri* | Kuankuoshui, Suiyang, Guizhou, China (30) | XM3393 | H24 | B21 | JX629518 | JX629548 | JX629623 | This study |
| *Q. boulengeri* | Kuankuoshui, Suiyang, Guizhou, China (30) | XM3402 | — | B16 | — | — | JX629622 | This study |
| *Q. boulengeri* | Kuankuoshui, Suiyang, Guizhou, China (30) | XM3395 | — | B17 | — | — | JX629624 | This study |
| *Q. boulengeri* | Kuankuoshui, Suiyang, Guizhou, China (30) | XM3396 | — | B21 | — | — | JX629625 | This study |
| *Q. boulengeri* | Kuankuoshui, Suiyang, Guizhou, China (30) | XM3397 | — | B22 | — | — | JX629626 | This study |
| *Q. boulengeri* | Kuankuoshui, Suiyang, Guizhou, China (30) | XM3398 | — | B2 | — | — | JX629627 | This study |
| *Q. boulengeri* | Kuankuoshui, Suiyang, Guizhou, China (30) | XM3399 | — | B21 | — | — | JX629628 | This study |
| *Q. boulengeri* | Kuankuoshui, Suiyang, Guizhou, China (30) | XM3400 | — | B20 | — | — | JX629629 | This study |
| *Q. boulengeri* | Kuankuoshui, Suiyang, Guizhou, China (30) | XM3401 | — | B17 | — | — | JX629630 | This study |
| *Q. boulengeri* | Leigongshan, Guizhou, China (31) | XY64 | H28 | B15 | JX629476 | JX629549 | JX629631 | This study |
| *Q. boulengeri* | Leigongshan, Guizhou, China (31) | XM3389 | H28 | B15 | JX629474 | JX629550 | JX629632 | This study |
| *Q. boulengeri* | Leigongshan, Guizhou, China (31) | XM3390 | H28 | B15 | JX629475 | JX629551 | JX629633 | This study |
| *Q. boulengeri* | Xuefengshan, Hunan, China (32) | XM3543 | H21 | — | JX629469 | JX629565 |  | This study |
| *Q. boulengeri* | Xuefengshan, Hunan, China (32) | XM3538 | H27 | B13 | JX629477 | JX629563 | JX629645 | This study |
| *Q. boulengeri* | Xuefengshan, Hunan, China (32) | XM3546 | H27 | B13 | JX629480 | JX629564 | JX629649 | This study |
| *Q. boulengeri* | Xuefengshan, Hunan, China (32) | XM3552 | H29 | B14 | JX629483 | JX629562 | JX629646 | This study |
| *Q. boulengeri* | Xuefengshan, Hunan, China (32) | XM3544 | H29 | B14 | JX629478 | JX629556 | JX629647 | This study |
| *Q. boulengeri* | Xuefengshan, Hunan, China (32) | XM3545 | H29 | B14 | JX629479 | JX629557 | JX629648 | This study |
| *Q. boulengeri* | Xuefengshan, Hunan, China (32) | XM3548 | H29 | B14 | JX629481 | JX629559 | JX629650 | This study |
| *Q. boulengeri* | Xuefengshan, Hunan, China (32) | XM3549 | H29 | B14 | JX629482 | JX629558 | JX629651 | This study |
| *Q. boulengeri* | Xuefengshan, Hunan, China (32) | XM3550 | H29 | B14 | JX629484 | JX629561 | JX629652 | This study |
| *Q. boulengeri* | Xuefengshan, Hunan, China (32) | XM3551 | H29 | B14 | JX629485 | JX629560 | JX629653 | This study |
| *Q. boulengeri* | Hejiapingzhen, Changyang Co., Hubei, China (33) | XM3560 | H19 | B8 | JX629488 | JX629521 | JX629581 | This study |
| *Q. boulengeri* | Hejiapingzhen, Changyang Co., Hubei, China (33) | XM3561 | H20 | — | JX629473 | JX629520 |  | This study |
| *Q. boulengeri* | Hejiapingzhen, Changyang Co., Hubei, China (33) | XM3559 | — | B2 | — | — | JX629580 | This study |
| *Q. boulengeri* | Hejiapingzhen, Changyang Co., Hubei, China (33) | XM3562 | — | B2 | — | — | JX629582 | This study |
| *Q. boulengeri* | Hejiapingzhen, Changyang Co., Hubei, China (33) | XM3564 | — | B19 | — | — | JX629583 | This study |
| *Q. boulengeri* | Hejiapingzhen, Changyang Co., Hubei, China (33) | XM3565 | — | B18 | — | — | JX629584 | This study |
| *Q. boulengeri* | Hejiapingzhen, Changyang Co., Hubei, China (33) | XM3566 | — | B8/B9/B10/B12 | — | — | JX629585 | This study |
| *Q. spinosa* | Jinhua, Zhejiang Prov, China | — | — | B1 | — | — | FJ432700 | Zhou et al., 2009 |
| *Q. boulengeri* | Chongqing, Youyang, China | — | — | B5 | — | — | JN700887 | Che et al., 2012 |
| *Q. robertingeri* | Hejiang Co., Sichuan, , China | — | — | B6 | — | — | JN700886 | Che et al., 2012 |
| *Nanorana cf. delacouri* 2 | Con Cuong Dist, Nghe An, Vietnam | — | H8 | — | EU979750 | EU979810 | — | Che et al., 2009 |
| *N. cf. delacouri* 1 | Kaleum Dist, Xe Kong Prov, Lao PDR | — | H7 | — | EU979752 | EU979812 | — | Che et al., 2009 |
| *N. medogensis* 2 | Medôg Co., Xizang, China | — | H2 | — | DQ118462 | DQ118506 | — | Che et al., 2009 |
| *N. unculuanus* 2 | Jingdong Co., Yunnan, China | — | H1 | — | DQ118447 | DQ118491 | — | Che et al., 2009 |
| *N. ventripunctata* 3 | Zhongdian Co., Yunnan, China | — | H3 | — | DQ118457 | DQ118501 | — | Che et al., 2009 |
| *Q. boulengeri* 1 | Mt. Emei, Sichuan, China | — | H14/H15/H17 | — | DQ118433 | DQ118477 | — | Che et al., 2009 |
| *Q. boulengeri* 10 | Lichuan, Hubei, China | — | H14/H15/H17 | — | EU979756 | EU979816 | — | Che et al., 2009 |
| *Q. boulengeri* 2 | Hunan, China | — | H22/H23 | — | EU979761 | EU979821 | — | Che et al., 2009 |
| *Q. boulengeri* 3 | Maolan nature reserve, Guizhou, China | — | H29 | — | EU979760 | EU979820 | — | Che et al., 2009 |
| *Q. boulengeri* 4 | Longqing, Shizong Co., Yunnan, China | — | H25 | — | EU979759 | EU979819 | — | Che et al., 2009 |
| *Q. boulengeri* 5 | Longqing, Shizong Co., Yunnan, China | — | H26 | — | DQ118435 | DQ118479 | — | Che et al., 2009 |
| *Q. boulengeri* 7 | Yihuang, Jiangxi, China | — | H22/H23 | — | EU979757 | EU979817 | — | Che et al., 2009 |
| *Q. boulengeri* 8 | Yichang, Hubei, China | — | H20 | — | EU979755 | EU979815 | — | Che et al., 2009 |
| *Q. boulengeri* 9 | Yichang, Hubei, China | — | H20 | — | EU979758 | EU979818 | — | Che et al., 2009 |
| *Q. cf. boulengeri* 6 | Tam Dao, Vinh Phu Prov, Vietnam | — | H12 | — | EU979791 | EU979851 | — | Che et al., 2009 |
| *Q. exilispinosa* 1 | Sangang, Wuyi, Fujian, China | — | H10 | — | DQ118440 | DQ118484 | — | Che et al., 2009 |
| *Q. jiulongensis* 1 | Sangang, Wuyi, Fujian, China | — | H9 | — | DQ118441 | DQ118485 | — | Che et al., 2009 |
| *Q. robertingeri* 1 | Zihuai, Hejiang Co., Sichuan, China | — | H13 | — | EU979754 | EU979814 | — | Che et al., 2009 |
| *Q. robertingeri* 2 | Zihuai, Hejiang Co., Sichuan, China | — | H13 | — | DQ118434 | DQ118478 | — | Che et al., 2009 |
| *Q. shini* 1 | Huaping, Longsheng Co., Guangxi, China | — | H6 | — | DQ118442 | DQ118486 | — | Che et al., 2009 |
| *Q. sp.* | Kaleum Dist, Xe Kong Prov, Lao PDR | — | H4 | — | EU979743 | EU979803 | — | Che et al., 2009 |
| *Q. spinosa* 2 | Mt.Dawei, Pingbian Co., Yunnan, China | — | H11 | — | DQ118437 | DQ118481 | — | Che et al., 2009 |
| *Q. verrucospinosa* 3 | Tam Dao, Vinh Phu Prov, Vietnam | — | H12 | — | EU979753 | EU979813 | — | Che et al., 2009 |
| *Q. yei* 1 | Shangcheng Co., Henan, China | — | H5 | — | DQ118444 | DQ118488 | — |  |
| * All rRNA+COI haplotypes contained the 12S and 16S sequences, and all COI+rRNA haplotypes contained the COI sequence. | | | | | | | | |
