# Supplementary material for: A De Novo Case of Floating Chromosomal Polymorphisms by Translocation in Quasipaa boulengeri (Anura, Dicroglossidae)
Source: PLoS One. 2012 Oct 3;7(10):e46163. doi: 10.1371/journal.pone.0046163 (PMC3463521; doi:10.1371/journal.pone.0046163)
Supplement: Table S2 — Primers used in PCR and sequencing of Quasipaa boulengeri in this study. (DOC) [file pone.0046163.s002.doc]

| Table S2 Primers used in PCR and sequencing of *Quasipaa boulengeri* in this study | | | |
| --- | --- | --- | --- |
| Locus | Primer | Primer sequence | Cited source |
| 12S rRNA | 12S-2_L1091 | 5'-AAAAAGCTTCAAACTGGGATTAGATACCCCACTAT-3' | Che et al., 2009 |
|  | 12S-2_H1478 | 5'-TGACTGCAGAGGGTGACGGGCGGTGTGT-3' | Che et al., 2009 |
|  |  |  |  |
| 16S rRNA | 16S L02510 | 5'-CGCCTGTTTATCAAAAACAT-3' | Che et al., 2009 |
|  | 16S H03063 | 5'-CTCCGGTTTGAACTCAGATC-3' | Che et al., 2009 |
|  |  |  |  |
| COI | VR1d | 5'-TAGACTTCTGGGTGGCCRAARAAYCA-3' | Ivanova et al., 2006 |
|  | VF1d | 5'-TTCTCAACCAACCACAARGAYATYGG-3' | Ivanova et al., 2006 |
