# Supplementary material for: A De Novo Case of Floating Chromosomal Polymorphisms by Translocation in Quasipaa boulengeri (Anura, Dicroglossidae)
Source: PLoS One. 2012 Oct 3;7(10):e46163. doi: 10.1371/journal.pone.0046163 (PMC3463521; doi:10.1371/journal.pone.0046163)
Supplement: Table S3 — Models selected for data partitions by the Bayesian information criterion (BIC) and corrected Akaike information criterion (AICc) * All rRNA+COI haplotypes contained the 12S and 16S sequences, and all COI+rRNA haplotypes contained the COI sequence. (DOC) [file pone.0046163.s003.doc]

| Table S3 Models selected for data partitions by the Bayesian information criterion (BIC) and corrected Akaike information criterion (AICc) | | | |
| --- | --- | --- | --- |
| data set* | partition | model (BIC) | model (AICc) |
| rRNA+COI | 12S | TIM2ef+G | TIM2ef+G |
|  | 16S | TPM1+I+G | TVMef+I+G |
|  | COI | HKY+G | HKY+G |
| COI+rRNA | COI | TrN+G | TIM3+G |
|  | 12S | JC | HKY |
|  | 16S | K80 | HKY |
| * All rRNA+COI haplotypes contained the 12S and 16S sequences, and all COI+rRNA haplotypes contained the COI sequence | | | |
